# WISEST: Weighted Interpolation for Synthetic Enhancement Using SMOTE with Thresholds †

**DOI:** 10.3390/s25247417

**Published:** 2025-12-05

**Authors:** Ryotaro Matsui, Luis Guillen, Satoru Izumi, Takuo Suganuma

**Affiliations:** 1 Graduate School of Information Sciences, Tohoku University, Aramaki Aza Aoba 6-3-09, Aoba-ku, Sendai 980-8579, Miyagi, Japan; 2 Department of Research, Science and Technology, Universidad San Francisco Xavier de Chuquisaca, Junin Esq. Estudiantes, Sucre, Chuquisaca, Bolivia; 3 National Institute of Technology, Sendai College, 4-16-1 Ayashi-Chuo, Aoba-ku, Sendai 989-3128, Miyagi, Japan; 4 Cyberscience Center, Tohoku University, 6-3, Aramaki Aza Aoba, Aoba-ku, Sendai 980-8578, Miyagi, Japan

**Keywords:** imbalanced datasets, class imbalance, oversampling, synthetic data generation, weighted interpolation, SMOTE, Borderline-SMOTE, locality-aware oversampling, WISEST, KEEL

## Abstract

**Highlights:**

**What are the main findings?**

- Consistent recall and F1 gains: WISEST increased recall and F1 across multiple benchmarks by generating weighted locality-constrained synthetic minority samples that reduce unsafe interpolation.
- Robustness to moderate noise and borderline structure: WISEST outperformed or matched alternatives when sufficient samples formed borderline pockets or subclusters, delivering stronger minority detection with modest precision trade-offs.

**What are the implications of the main findings?**

- The approach is a practical choice for recall-critical applications: for tasks where detecting minority events matters (e.g., intrusion detection and rare-fault detection), WISEST offers a reliable oversampling option without aggressive sample generation or complex training.
- There is no “one oversampler that rules them all” for imbalanced datasets: WISEST should be used alongside dataset diagnostics. Datasets with limited minority support, extreme separability, highly noisy borderlines, or many categorical features may still benefit more from other methods.

**Abstract:**

Imbalanced learning occurs when rare but critical events are missed because classifiers are trained primarily on majority-class samples. This paper introduces WISEST, a locality-aware weighted-interpolation algorithm that generates synthetic minority samples within a controlled threshold near class boundaries. Benchmarked on more than a hundred real-world imbalanced datasets, such as KEEL, with different imbalance ratios, noise levels, geometries, and other security and IoT sets (IoT-23 and BoT–IoT), WISEST consistently improved minority detection in at least one of the metrics on about half of those datasets, achieving up to a 25% relative recall increase and up to an 18% increase in F1 compared to the original training and other approaches. However, in most cases, WISEST’s trade-off gains are in accuracy and precision depending on the dataset and classifier. These results indicate that WISEST is a practical and robust option when minority support and borderline structure permit safe synthesis, although no single sampler uniformly outperforms others across all datasets.

## 1. Introduction

Machine learning (ML) and Artificial Intelligence (AI) have recently become widespread, helping to create robust solutions across different fields from medical care [1] to cyberattack detection [2]. ML systems require high-quality datasets for learning or training. However, not all datasets possess high-quality/representative data, and the sample distributions are often unbalanced. The issue with imbalanced datasets, which are structured to favor a majority class, is that, when ML models are trained on them, the apparent accuracy is high. However, they cannot detect anomalies because most of the training data comes from the majority class. Consequently, misclassification is unavoidable [3], thereby decreasing detection precision, especially at the boundary between classes.

The traditional approach to handling imbalanced datasets is by “oversampling”. Oversampling increases the number of samples from the minority class, typically using algorithms such as SMOTE [4] and ADASYN [5]. These methods increase the number of instances in the minority class. However, they are created at random locations, even in areas where it might increase the chance of misclassification, that is, at the borderline between the majority and minority classes. Approaches such as Borderline-SMOTE [6], SMOTEENN [7], or WCOM-KKNBR [8] worked on strategies to overcome the imbalance issue by applying innovative and effective solutions. However, all these traditional and emerging works highlight two points. First, the class imbalance problem is still an open issue, and, second, no single method is universally the “best” for all situations. In fact, to the best of our knowledge, there are no existing methods to increase the minority-class count without generating extra noise at the borderline, or that consider various dataset elements, such as geometry or local class overlaps.

In our prior work [9], we designed a basic algorithm that considered the elements above; that is, a locally aware minority-class oversampling method at the borderline, thereby avoiding unsafe synthetic generation that would harm precision. However, the evaluation was primarily conducted on synthetic datasets, which may not reflect actual performance on real-world datasets. Therefore, this paper enhances our initial proposal, and its main contributions are threefold:

- We present WISEST, an oversampling algorithm that assigns weights to near neighbors and interpolates synthetic samples using SMOTE within a threshold.
- We extensively test WISEST against traditional and oversampling methods using real-world datasets.
- Finally, we present a thorough analysis of the conditions by which WISEST performs better than existing work.

Based on our results across various real-world imbalanced datasets, we concluded that, when the minority class has a moderate number of samples and is not isolated (i.e., it has both minority and majority neighbors nearby), WISEST can interpolate within local neighborhoods to increase recall without creating many outliers. Thus, its per-sample weighting avoids oversampling dense subclusters and instead enhances sparser border regions, which helps models to detect minority sub-classes more reliably. Therefore, if F1 or recall is the evaluation metric, WISEST commonly yields the best scores in more than half of the datasets tested.

Before delving into the (proposed) WISEST oversampling method presented in Section 3 and the idea behind it, we describe the related work on traditional and modern oversampling methods in Section 2. Section 4 presents and discusses the benchmark results from extensive testing. Finally, Section 5 concludes this paper with a brief summary of our findings and future lines of work.

## 2. Related Work

SMOTE [4] is the simplest and most common method for oversampling imbalanced datasets. In brief, new synthetic samples are generated between two randomly selected minority-class points until the number of samples equals the majority class. This process creates unnecessary samples without accounting for the dataset’s distribution or geometry. ADASYN [5], an extended version of SMOTE, unlike SMOTE, does take into account the density distribution [10]. Still, the aggressive sampling remains the same as with SMOTE; moreover, it is limited to five dimensions [11]. Finally, Borderline-SMOTE [6] improves SMOTE by targeting the area between the majority and minority classes. In brief, a new synthetic sample is created in a random location among *k* neighboring minority samples closer to the majority class. Thus, although it restricts the area by concentrating on borderline points, it can also amplify ambiguous or mislabeled instances and increase false positives if boundaries are noisy.

Beyond the traditional oversampling algorithms presented above, other interesting approaches have improved SMOTE. For instance, Dai et al. [12], Radius-SMOTE [13], or SMOTEENN (SMOTE Edited Nearest Neighbor) [7] focus on “where” to create the new synthetic data by using distance-based oversampling near the minority class. These methods are effective for specific cases but still have limitations. For instance, SMOTEENN has a “cleaning step” that increases the computation and might remove functional minority samples. Radius-SMOTE requires careful radius/neighbor settings that are less aggressive than those of other methods, but it suffers considerable degradation on noisy boundaries. SMOTE+Tomek [14], similarly to SMOTEENN, is a two-step resampling pipeline that first oversamples the minority class with synthetic samples and then cleans the class boundary by removing Tomek links (nearest-neighbor pairs from opposite classes, typically deleting the majority member). However, as with the other SMOTE-based approaches above, it can produce unrealistic or noisy synthetic samples (especially near class boundaries, in sparse or high-dimensional data, or across distinct minority modes), which may cause overfitting. Moreover, Tomek link removal can discard informative majority examples and is sensitive to distance metrics and scaling; together, they require careful preprocessing and tuning to avoid amplifying outliers or harming generalization.

Other approaches, such as SMOTE-WENN [15] or WCOM-KKNBR [8], focus on “how” to address the imbalance using a weighted approach with either heuristics [15] or, more recently, machine- and deep learning [8] techniques. However, their strategies still have limitations. SMOTE-WENN still introduces noisy/borderline synthetic samples and overfits in small or high-dimensional minority pockets. At the same time, WCOM-KKNBR’s training remains unstable because it does not account for the dataset’s geometry.

Therefore, this paper presents a robust hybrid method that combines locality-aware safety with generative fidelity for imbalanced datasets, thereby filling the gap in the above work.

## 3. Proposed WISEST Oversampling

### 3.1. Overview

**Figure 1:**
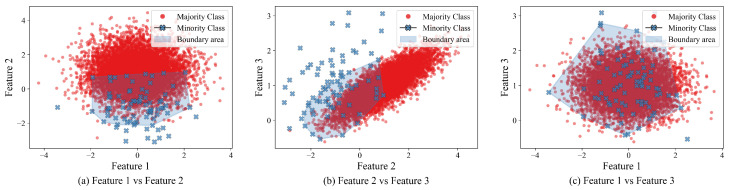
Feature pairwise scatterplots from a synthetic dataset with their boundary area highlighted in shaded blue.

This section details the proposed oversampling method, WISEST (Weighted Interpolation for Synthetic Enhancement using SMOTE with Thresholds). The main features can be summarized as follows:

1. WISEST creates minority synthetic samples in the “boundary area”, which is located between the majority and minority classes. This will avoid excessive and indiscriminate creation of samples that might hinder classification. For illustration purposes, we created a synthetic binary classification dataset using the following code:
make_classification(n_samples=10000, n_features=3,n_redundant=0,n_clusters_per_class=1, weights=[0.99],flip_y=0,random_state=1)
  The make_classification function from the sklearn.datasets library in Python 3.10.15 creates n_samples=10000 samples, with three non-redundant features (independent variables); the minority and majority classes will consist of a single cluster with no noise, and the IR (IR) would be 99:1. That is, 1% of the samples would belong to the minority class.
  Figure 1 depicts the generated dataset (majority class samples depicted in red; minority in blue) and the boundary area (shown in shaded blue) from a synthetic dataset created above. Note that the area varies depending on the features being compared and the dataset’s geometry.
2. Some feature pairs might have high separability (i.e., Figure 1b), while others might have overlapping classes (i.e., Figure 1c). Thus, we apply a weighted locally aware interpolation within a threshold. To do so, we use a custom threshold distance to limit the area around each nearest minority class. This distance is used to assign weights to each minority point. Then, we apply conditional branching based on this weight; thus, even when the minority class data is sparsely distributed in the dataset, we can generate samples from the closest minority neighbors.
3. Contrary to traditional approaches, such as SMOTE, ADASYN, and Borderline-SMOTE, which create the same number of samples as the majority-class data, we adopt a more conservative approach, producing only samples that fall within the boundary area, thereby avoiding over-inflating the dataset with samples that might affect its performance.

### 3.2. The WISEST Algorithm

Algorithm 1 presents a pseudocode of the proposed WISEST oversampling based on the premises outlined in Section 3.1. As the first step, we calculate the weight (*w*) for each data point in the minority class, which is determined by the ratio of the number of points that belong to the majority class (NN) among the *k* neighboring points, as shown in Equation (1).

(1) $$w_{i}=\frac{NN}{k}\;i:1,2,\ldots ,k$$

**Algorithm 1.** Proposed Algorithm  1:**Input:** Training dataset *T*, Threshold θ  2:**Output:** Resampled dataset  3:**for** each minority class sample *i* in *T* **do**  4:      Find its *k* nearest neighbors in *T*  5:      NN← number of neighbors from the minority class  6:      $w_{i}\leftarrow \frac{NN}{k}$  7:      **if** $w_{i}<1$ **then**                                          ▹ No majority samples near or less than *k*.  8:            do nothing  9:      **else if** $w_{i}=1$ **then**                            ▹ Exactly the minimum number of neighbors.10:            **for** each neighbor *j* **do**                                                                        ▹j=1,2,…,k11:                  Compute distance $dif_{ij}$ to nearest minority neighbor12:                  **if** $dif_{ij}\leq \theta$ **then**13:                        Generate sample $t^{\prime }$ using SMOTE in range 0≤r≤θ14:                  **end if**15:            **end for**16:                  **else**                                                   ▹ There are enough majority neighbors17:            Calculate the number neighbors *n* in range $0<j^{\prime }<j$18:            **for** each neighbor *j* **do**                                                                        ▹j=1,2,…,n19:                  Compute distance $dif_{ij}^{r}$ to nearest minority neighbor 0≤r≤θ20:                  Compute distance $dif_{ij}^{s}$ to nearest majority neighbor 0≤s≤θ/221:                  **if** $dif_{ij}^{r}\leq dif_{ij}^{s}$ **then**22:                        Generate $t^{\prime }$ samples using SMOTE in range 0≤r≤θ    ▹ Wider range.23:                  **else**24:                        Generate $t^{\prime }$ samples using SMOTE in range 0≤s≤θ/2    ▹ Less range.25:                  **end if**26:                  Add $t^{\prime }$ to *T*27:            **end for**28:      **end if**29:**end for**30:**return** *T*

As the next step, WISEST applies conditional branching using the following criteria:

- When no majority-class points (NN) exist nearby the *k* neighboring points (measured by the distance *dif*) that are within the threshold (θ), then no synthetic data is generated as it would not be within the boundary area, as detailed in lines 7 and 8 in Algorithm 1.
- Now, when the ratio of the number of majority and the selected *k* minority samples is equal, that is, there are only the minimum number of majority samples (NN) equal to *k*, then w=1. This case is treated separately as the dataset may be very sparse or contain an isolated boundary. Thus, we adopt a conservative approach and create only *k* synthetic data toward the minority class. To do so, we calculate whether the distance is within the threshold (dif≤θ) and whether the distance to the closest minority (*r*) neighbor is 0≤r≤θ using SMOTE, as shown in lines 9 to 15 in Algorithm 1.
- Otherwise, since there are enough majority-class neighbors, we must first determine the number of samples to create (*n*); we do this based on the number of nearest minority samples, as shown in Equation (2):
  (2) $$n=\frac{w_{j}\;\cdot \;n_{j}^{\prime }}{w_{j}^{\prime }}\;in\;range\;0<j^{\prime }<j$$
  Then, *n* synthetic samples are generated in the range 0≤r≤θ if the distance from the sample to the closest minority neighbor is less than or equal to the closest majority neighbors (*s*), otherwise within the range 0≤s≤θ/2 of the majority class, as described in Algorithm 1, lines 17 to 29. The rationale for this is that we prefer to create samples near the minority class in cases where it is closer but not so close to the majority neighbor.

### 3.3. WISEST Applied on the Synthetic Dataset

**Figure 2:**
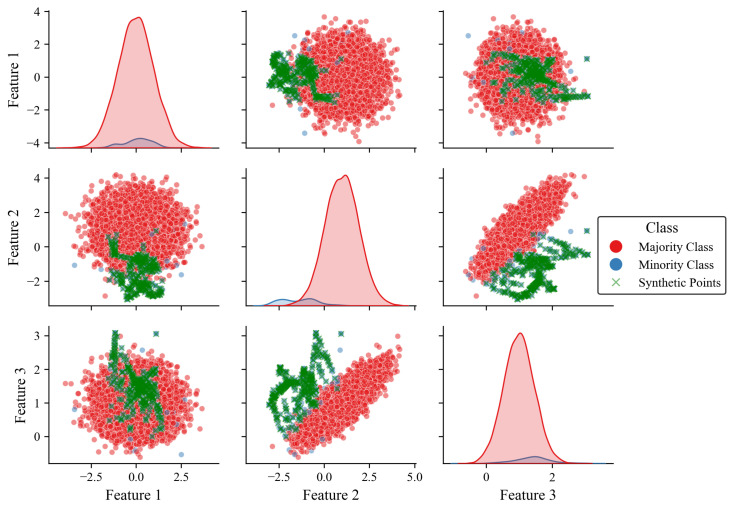
Pairplot of features 1 to 3 with classes oversampled by our WISEST approach. Note the shaded areas represent the imbalance between the majority and minority classes.

To illustrate how WISEST works, Figure 2 depicts the results of applying our proposed oversampling to the synthetic dataset presented in Section 3.1 and its corresponding class distribution. Note that WISEST generates synthetic points within the threshold-defined boundary, with minimal outlier samples.

### 3.4. Preliminary Analysis of Oversampling Methods on the Synthetic Dataset Compared to WISEST

**Figure 3:**
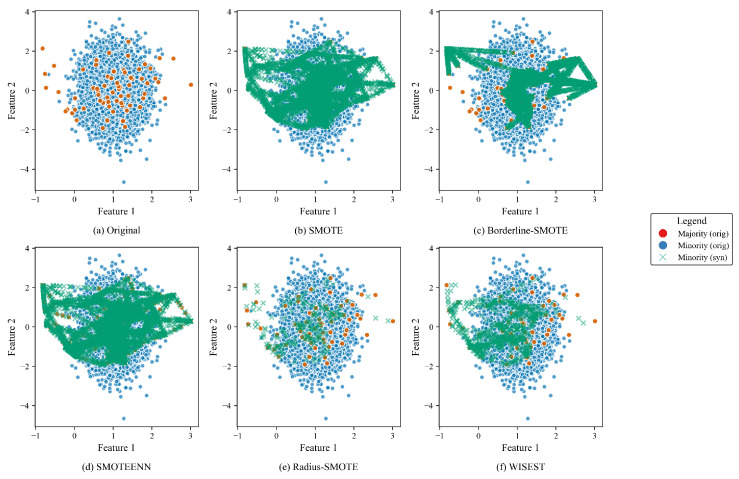
Comparative visualization of oversampling methods for features 1 and 2.

As a preliminary experiment, we run other algorithms, both traditional (SMOTE [4] and Borderline-SMOTE [6]) and existing work (SMOTEENN [7] and Radius-SMOTE [13]), on the same synthetic dataset presented in Section 3.1. For brevity, we only considered two features (i.e., feature 1 vs. feature 2). The goal of this preliminary evaluation is to (visually) assess how different strategies produce minority synthetic samples and compare them with the original. Figure 3 shows how SMOTE and SMOTEENN aggressively populate the samples all over the dataset. Borderline-SMOTE is a less intensive approach, but it still may cause misclassification in some areas. On the other hand, Radius-SMOTE and WISEST present the most conservative approaches.

**Figure 4:**
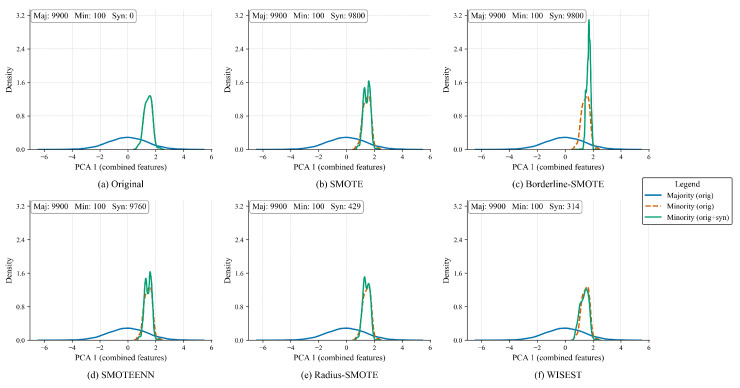
Comparative density analysis of oversampling methods in PCA-1 projection showing the distributional impact in the minority class.

Additionally, we conducted a Kernel Density Estimate (KDE) analysis of the effects of each method on the number of generated samples and the distribution of the dataset. To do so, we performed a Principal Component Analysis (PCA) to reduce the dataset’s dimensionality. The PCA-1 projections of the original and oversampled datasets are shown in Figure 4. The legend at the top shows the synthetic samples generated per approach.

As observed, the original dataset, shown in Figure 4a, is composed of 9900 majority and 100 minority samples and presents a clear distribution between classes. While oversampling the datasets with SMOTE (Figure 4b), Borderline-SMOTE (Figure 4c), and SMOTEENN (Figure 4d) produces almost the same amount of minority samples as the majority, note that the newly produced samples alter the minority distribution. On the other hand, Radius-SMOTE (Figure 4e) and WISEST (Figure 4f) adopt a more conservative approach, producing around 5% of the results of the others.

However, note that, among the tested approaches, WISEST alters the original distribution the least; thus, this might help to preserve the original dataset’s accuracy while creating only “safe” samples. Of course, being a straightforward synthetic dataset, this might not hold in real-world datasets. Therefore, in the next section, we present a thorough evaluation of these approaches, considering formal performance metrics and settings.

## 4. Evaluation and Discussion

This section benchmarks WISEST against existing traditional and state-of-the-art oversampling algorithms and discusses its implications.

### 4.1. Experimental Setup

#### 4.1.1. Datasets

We used two groups of well-known real-world datasets: KEEL [16] and other IoT/security-related datasets (IoT-23 [17], BoT–IoT [18], and Air Quality and Pollution Assessment [19]).

The KEEL repository comprises a variety of imbalanced datasets, as detailed in Table 1. Note that we have only included the binary class datasets and subdivided the tests depending on the IR as follows:

- Datasets with an IR between 1.5 and 9: 20 dataset subgroups (e.g., iris, glass, pima, new-thyroid, vehicle, some yeast subset variants, some ecoli subset variants, and shuttle).
- Datasets with an IR higher than 9: 70 dataset subgroups (e.g., some abalone subsets, winequality subsets, poker class-pair problems, KDD attack pairs, some ecoli subsets, and some yeast subset variants).
- Noisy and borderline examples: 30 dataset subgroups (e.g., 03subcl5, 04clover5z, paw02a families and their noise levels variants).

The other datasets contained multiple class imbalances, detailed as follows:

- IoT-23 [17] contains data on attacks on IoT devices such as Amazon Echo. The dataset is classified into six classes with an IR of 57:18:14:10:1:0.2. We used the following columns: orig_bytes, orig_okts, orig_ip_bytes, resp_bytes, resp_pkts, and resp_ip_bytes, and the classification labels were PortOfHorizontalPortScan, Okiru, Benign, DDoS, and C&C. Note, we only used the first 10,000 samples for both training (80%) and testing (20%).
- BoT–IoT [18] is a dataset from a realistic network environment that classifies the network data into four classes: normal, backdoor, XSS, and scanning, with an IR of 84:14:1.8:0.2. We used the following columns: FC1_Read_Input_Register, FC2_Read_Discrete_Value, FC3_Read_Holding_Register, and FC4_Read_Coil. We used the same number of samples and distributions as with IoT-23.
- Air Quality and Pollution Assessment [19] is a dataset containing environmental and demographics data regarding air quality, which is separated into four classes (Good, Moderate, Poor, and Hazardous), with an IR of 4:3:2:1. We used the Temperature, Humidity, PM2.5, PM10, NO_2_, SO_2_, CO, Promixity_to_Industrial_Areas, and Population_Density columns and 5000 samples.

Note that the datasets above differ in terms of IR, sample distributions (e.g., borderline and noise levels), and class separability. This variety allowed thorough testing.

#### 4.1.2. Oversampling Techniques

We used the following approaches to benchmark WISEST:

1. SMOTE [4]
  Base technique that generates a new minority-class sample using linear interpolation between a minority sample and one of its *k* nearest minority neighbors.
2. Borderline-SMOTE [6]
  A variant of SMOTE that creates synthetic samples near the class boundary instead of between minority points.
3. ADASYN [5]
  Generates more synthetic minority samples near hard-to-learn samples, so classifiers focus on difficult regions.
4. SMOTE+Tomek [14]
  Creates synthetic minority samples (SMOTE) and then removes overlapping borderline pairs (Tomek links) to balance and clean the data.
5. SMOTEENN [7]
  An approach that combines SMOTE with Edited Nearest Neighbor (ENN), which removes samples whose labels disagree with the majority of their *k* nearest neighbors.
6. Radius-SMOTE [13]
  Another SMOTE variant that restricts or selects interpolation inside a fixed neighborhood radius, which reduces unsafe extrapolation and limits generation in sparse/noisy regions.
7. WCOM-KKNBR [8]
  Uses conditional Wasserstein CGAN-based oversampling, which generates minority class samples to balance the majority using a K-means and nearest neighbor-based method.
8. WISEST
  Our approach.

These approaches were selected because, except for SMOTE, they generate synthetic samples in regions near the borders between the majority and minority classes as WISEST does.

#### 4.1.3. Implementation and Setup

SMOTE, BorderlineSMOTE, ADASYN, SMOTE+Tomek, and SMOTEENN were already implemented into the imblearn library in Python, while Radius-SMOTE, WCOM-KKNBR, and our algorithm were implemented using Python 3.10.15 in a locally deployed Jupyter Notebook version 7.4.6. The experiments were performed on a MacBook Pro with a silicon M1 Max Chip with 30 GPU cores and 64 GB of memory.

#### 4.1.4. Methodology

The datasets with nominal variables (e.g., KDD variants and Poker) were preprocessed by converting them to numerical values using Dummy Encoding. The feature selection used for the evaluation comprised a five-fold cross-validation to estimate variance with the same random number generator (RNG) as a seed (RNG=42) for all algorithms.

The common and specific parameters used for each algorithm are described below:

- SMOTE: k=5 neighbors. The rationale for this number was to set “enough” NNs to ensure the newly generated samples would safely lie within the boundary area. Based on preliminary experiments, it was determined to be the optimal value. However, we will address a thorough analysis of how this value affects each dataset in the future.
- Borderline-SMOTE: variant 1, k=5 neighbors.
- ADASYN: Same as SMOTE, sampling adaptivity enabled.
- SMOTE+Tomek: SMOTE component same as above, and Tomek link step has no tunable hyperparameter.
- SMOTEENN: Same SMOTE parameters as above, ENN=k=5.
- Radius-SMOTE: As defined in [13].
- WCOM-KKNBR: latent dimension latent_dim = 16; epochs = 50; batch_size = 32; learning rates $\eta_{G}=2\times 10^{-4}$ (generator) and $\eta_{D}=1\times 10^{-4}$ (discriminator); generate $n_{\mathrm{samples}}$ equal to the original minority count, as defined in [8].
- WISEST (proposed): k=5 neighbors and threshold distance θ=0.5. Note that the threshold value has been statically set based on preliminary experiments with the synthetic dataset shown in Section 3.1.

Once the datasets were resampled, we generated synthetic sample counts for each method. Then, we classified the newly resampled dataset using Random Forest. Finally, the benchmark metrics (precision, recall, accuracy, and F1) were calculated for both the original and resampled datasets.

We conducted the above procedure for each category of the KEEL datasets described in Section 4.1.1. The experiments were run on datasets with IR of less than 9 and greater than 9, as well as variants with controlled noise levels.

### 4.2. Results

#### 4.2.1. Results on KEEL Datasets with IR Less than or Equal to 9

In this section, we tested each benchmark method on real-world KEEL datasets with an IR of 9 or less (22 datasets in total).

First, we analyzed the number of synthetic samples generated per approach as a reference for how each dataset’s characteristics affect sample generation. Table 2 summarizes the number of synthetic points created per approach, where the lowest value is highlighted in bold as a reference.

As observed, our WISEST approach generated the fewest synthetic minority samples only on the ecoli-0_vs_1 dataset, while WCOM-KKNBR created the least in about 60% of the tested datasets. However, note that we do not intend to produce the smallest number but only those that are within *k* minority-class points of the boundary. Therefore, we analyzed the characteristics of the datasets for which WISEST generated the fewest and most synthetic samples, as detailed in Table 3.

According to the examined datasets, if the minority class has a high class overlap, the distance to the majority class at the borderline is low, i.e., low separability between classes, such is the case with the ecoli variants and yeast; WISEST produces fewer “unsafe” interpolation targets and therefore generates fewer new points. In contrast, if there is a distinct class separation or compact minority clusters, WISEST creates up to 5 times more points than its counterparts as these are considered “safe” to add, even when some exhibit low separability or imbalance.

Next, we measured the benchmark metrics (accuracy, precision, recall, and F1) for KEEL datasets with an IR less than 9, highlighting the highest value for each metric in bold. Table 4 shows the results of selected datasets where WISEST performed the best in at least one of the metrics. However, the full results are available in Appendix A.

Based on the above results, WISEST presents the best or comparable performance in the following datasets: glass1, ecoli-0_vs_1, iris0, yeast1, haberman, glass-0-1-2-3_vs_4-5-6, new-thyroid1, new-thyroid2, and glass6. The improvements were, on average, 8% in recall (peak at 16% on yeast1) and 3% in F1 (peak at 10% on haberman) compared to the original (relative), and about 2% compared to the other methods (relative). However, in the same datasets, the decrease in accuracy and precision was about 1 to 4% on average, respectively.

After running diagnostics on the characteristics of each dataset, we can conclude that, in datasets with a nontrivial fraction of minority points near class boundaries (i.e., glass1, yeast1, pima, haberman, glass-0-1-2-3_vs_4-5-6, and glass6), or if the minority class presents multiple small minority subclusters near majority modes (i.e., glass1, yeast1, glass-0-1-2-3_vs_4-5-6, glass6, vehicle1, and vehicle3), or moderate label noise or borderline examples (i.e., yeast1, haberman, pima, glass1, and glass6), WISEST performs well. That is almost two-thirds of the tested datasets. However, on the contrary, WISEST underperforms or is even rendered unnecessary when the datasets have either extremely low minority support (i.e., ecoli_0vs_1, ecoli1, ecoli2, ecoli3, and page-blocks0), or nearly-separable classes (i.e., glass0, vehicle2, vehicle0, and segment0), or the nearest-neighbor geometry highly depends on the distance metrics (e.g., ecoli* variants and page-blocks0), causing unsafe interpolation. Nevertheless, the difference from the best performers is not that far, even in these datasets.

Note that the number of newly created synthetic minority samples did not influence the performance. Take new-thyroid2, for example, where WISEST generated almost 4 times as many samples as the rest; however, it performed better across nearly all metrics.

To sum up, for datasets with IR less than 9, if a dataset pre-diagnostic shows a sizable frac_border_k (e.g., ≥0.15~0.4), mean minority to majority distances comparable to minority internal spacing, and you can afford a small precision drop (approx. 4%) for larger recall/F1 gains (about 10%), WISEST would be preferred against the existing approaches.

#### 4.2.2. Results on KEEL Datasets with an IR Greater than 9

Next, we benchmarked WISEST against existing methods using KEEL datasets with an IR greater than 9 (i.e., highly imbalanced). As with the prior case, we tested the influence of each oversampling method on both the number of synthetic samples and performance. From a total of 69, we could test in the KEEL dataset. Table 5 presents the minority synthetic count results. However, due to space constraints, we show only the top 10 datasets for which WISEST generated the fewest points, including ties, and the full results are in Appendix B. Note, we could not test all of those (i.e., winequality-white-9_vs_4, zoo-3, shuttle-c2-vs-c4, lymphography-normal-fibrosis, and kddcup-land_vs_portsweep) with very few total minority samples (sometimes single-digit minority counts) and very few (under 200) samples overall. Thus, most of the sampling algorithms did not execute.

Across the 69 datasets, WISEST produced the fewest synthetic samples in almost half of them. Among these datasets, there were variations among abalone, cleveland, ecoli, glass, poker, winequality (red and white), and yeast. Similarly to the prior experiment, we also analyzed the characteristics of randomly selected datasets in which WISEST created the fewest (and otherwise) synthetic samples, as shown in Table 6.

In contrast to the experiment in Section 4.2.1, the distance to the majority class at the borderline, or the IR, is not a determining factor. However, note that, in cases of high class granularity, such as with KEEL variants like various class-subset or one-vs.-rest variants (yeast, abalone, glass, and ecoli), where minority labels come from narrow subpopulations that are structurally distinct, WISEST produces fewer “unsafe” points and with more certainty (frac_with_majority_neighbor_k5=1).

On the other hand, WISEST produces the most synthetic samples, in which the minority class is distributed across many locally mixed (majority and minority) borderline neighborhoods, and expanding minority coverage at those boundaries is useful; this results in larger synthetic counts than bulk oversamplers that apply a uniform rule across the minority class.

Next, we measured the metrics (accuracy, precision, recall, and F1). Again, for space’s sake, Table 7 shows only the results where WISEST performs the highest or tied for highest in at least one metric. A complete list of all the datasets in this category can be found in Appendix C.

As observed, across all datasets tested in this category, WISEST performed best and achieved competitive values in more than half of the datasets (36 out of 69 in total). The best results were achieved in terms of recall and F1, which were the highest (or competitive) in various yeast variants, vowel0, glass-0-1-6_vs_5, ecoli variants, shuttle-c0_vs_c4, page-blocks-1-3_vs_4, dermatology-6, and others. The results yield a recall improvement of up to 25% (5% AVG) relative to the original; for F1, the improvements were up to 18% (4% AVG) relative to the classifier trained on the original dataset or different strategies.

As for the datasets’ characteristics, many are class-subset or one-vs.-rest KEEL variants (ecoli, yeast, abalone, and glass) with locally complex boundaries that benefit from targeted boundary-focused sampling, as in our approach. Therefore, if there is a moderate minority (not small), these are enough seeds for WISEST to generate local, useful, and diverse synthetics across boundary regions. With these results, we can confirm that WISEST usually trades small precision and accuracy (0–7% AVG, respectively, relative to the original or other approaches) for better minority detection (i.e., recall and F1). Also, we confirmed that, even though WISEST did not produce the fewest samples, it still achieved gains compared to other approaches unless the original performance was already high.

To sum up, in KEEL datasets with IR greater than 9, WISEST performs best when the minority class is distributed across many locally mixed neighborhoods (borderline points) and when recall/F1 are the metrics of focus. As evident in the expanded results, WISEST often ties with Radius-SMOTE and WCOM-KKNBR in various metrics or achieves slightly higher F1 when safe boundary-focused interpolation is needed. Furthermore, on perfectly separable datasets, WISEST ties with other methods, yielding no practical advantage except for the processing time compared to heavy-processing approaches, such as WCOM-KKNBR (CGAN-based).

#### 4.2.3. Results on KEEL’s Noisy and Borderline Datasets

The last set of experiments run on KEEL datasets tested tolerance to varying levels of noise. To this aim, KEEL provides preprocessed datasets that alter the base dataset (i.e., 03subcl5, 04clover5z, and paw02a) in the number of samples (i.e., 600 or 800), cluster setup (e.g., 5 or 7 parameters), and noise or borderline level (from 0 to 70%), making a total of 30 variations. For all variations, we used the binary-imbalanced version to ensure consistency with prior experiments. For instance, the dataset 03subcl5-600-5-30-BI is a binary variant of the 03subcl5 dataset family, generated with 600 cases, five parameters, and 30% noise or borderline examples.

Table 8 displays the benchmark results for all parameters (accuracy, precision, recall, and F1). However, once again for space’s sake, we only show the datasets where WISEST performs the best in any of the results; full results can be found in Appendix D.

The results show that WISEST improves the recall and in some cases F1-score in datasets with pronounced borderline structure and moderate noise levels (0–30%). However, it falls short compared to the others on datasets with noise levels above the 30% threshold. In datasets where minority points have enough nearby minority neighbors to permit safe interpolation (the 600–800 and 5–7 parameterized synthetic datasets), let WISEST generate useful localized synthetics. In those cases, WISEST’s weighted location-aware synthesis increases true-positive detection near decision boundaries without producing many unsafe samples, thereby lifting recall and, in turn, F1. In terms of recall, we observed up to 23% (13% AVG) relative to the original but −5% on AVG compared to others, especially WCOM-KKNB. For F1, improvements of up to 12% (3% AVG) relative to the original dataset and up to 10% (−1% AVG) relative to different strategies were observed. Regarding datasets with high or very high noise rates (50–70%), the policies applied by SMOTEENN or Borderline-SMOTE, which intensively clean the minority class, can improve precision. Surprisingly, WCOM-KKNB performed the best in noisy variants of paw02a dataset but not in other datasets. Nevertheless, WISEST remains conservative in this situation, creating fewer (but safer) synthetic points, which makes it perform lower compared to the others but remain competitive.

#### 4.2.4. Results on Other Datasets Using Different Classifiers

This section evaluated the performance of WISEST when training ML classifiers on datasets other than the KEEL datasets, for instance, IoT-23 [17], BoT–IoT [18], and Air Pollution [19]; in particular, we used K-Nearest Neighbor (KNN), Random Forest (RF), and LightGBM trained with 80% of the oversampled dataset for training and the rest for testing. The goal of this experiment was to observe the difference compared to the original dataset. Thus, as with the other experiments, we measured accuracy, precision, recall, and F1. Note that, since WISEST was conceived with binary-class datasets in mind, this experiment also serves to analyze its behavior in multi-class environments. Nevertheless, testing WISEST robustness on multi-class datasets would be a topic for a future work.

The results are summarized in Table 9. As observed, the oversampling strategy applied by WISEST consistently improved most ML models. For instance, in the IoT-23 dataset and BoT–IoT, KNN achieved up to 50% improvement over the original data, and LightGBM achieved about 15% improvement, especially in recall and F1. On the other hand, WISEST performed slightly worse when applied to the Air Quality and Pollution Assessment dataset, about 1–2% lower than the algorithms trained on the original data. Unlike BoT–IoT and IoT-23, which exhibit a moderate to extreme imbalance depending on the classes, many borderline samples resemble benign traffic. Air Quality and Pollution Assessment’s IR is 1:4 at maximum, with very low borderline examples at the threshold; thus, time-series methods or regression models would be more appropriate than classification models. As described in prior experiments, WISEST would create many samples when the classes’ separability is high, which decreased the performance by adding more minority samples to the centroid rather than the borderline.

### 4.3. Sensitivity Analysis of the Threshold Distance

**Figure 5:**
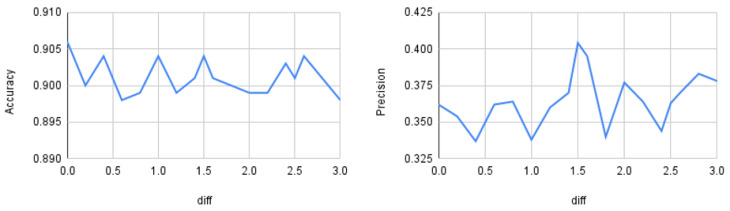
Quantitative differences as a function of the threshold distance. On the **left**: accuracy; on the **right**: precision.

**Figure 6:**
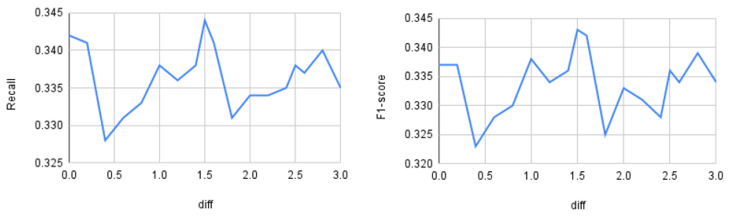
Quantitative differences as a function of the threshold distance. On the **left**: recall; on the **right**: F1-score.

Up to this point, the distance threshold (θ) was set using prior experiments on the synthetic dataset described in Section 3.1. This section evaluates how the input threshold affects model performance. We varied the distance parameter dif from 0 to 3 and measured accuracy, precision, recall, and macro-F1 on the BoT–IoT dataset using a Random Forest classifier. Figure 5 shows accuracy and precision, and Figure 6 shows recall and F1.

As observed, accuracy shows little variation across different dif values (approximately 0.5% relative change on average), whereas precision, recall, and macro-F1 vary more substantially (roughly 5–10% relative change), peaking at dif=1.5. Therefore, for the BoT–IoT dataset with a Random Forest classifier, the optimal threshold θ is 1.5. From this, we can conclude that it is important to perform similar preliminary analyses for other datasets and classifiers before running the oversampling procedure. However, dynamic threshold selection is left for future work.

### 4.4. Discussion

The prior sections described extensive testing across different datasets, from synthetic to real-world, with varying IR, types, contexts, and noise/borderline levels. From these results, we conclude that no single approach is suitable for all cases. Even vanilla SMOTE performed better than other methods for specific datasets under certain conditions.

WISEST uses a weighted interpolation approach to create synthetic samples within a threshold between the majority and minority classes, which has been shown to improve performance (especially recall and F1 measures) in various scenarios. For instance, as shown in Section 4.2.1–Section 4.2.3, when there is a significant fraction of minority samples with majority neighbors (borderline points), WISEST is designed to adapt sampling based on local class composition by targeting synthetic samples that safely expand minority coverage around boundaries, improving recall and F1. On the other hand, in very sparse minority structures, highly noisy borders, or clearly separable classes, WISEST will perform as if without the oversampling (i.e., original dataset) or will underperform compared to other alternatives, both SMOTE-based or otherwise.

Moreover, in Section 4.2.4, we showed that the WISEST oversampling strategy improved performance when used with multi-class imbalanced datasets. As with KEEL datasets, the number of borderline samples influenced precision, as expected in imbalanced datasets [20,21]. Note that, even if the IR differs for each pair of classes, WISEST uses a single (static) threshold across all the tested datasets in the current implementation, which is expected to yield better performance in the binary case. At the same time, the multi-class setup would require a more careful analysis for each pair of classes. Therefore, it is a limitation we plan to study and overcome as future work. However, we believe that dynamic threshold selection will make WISEST a more robust oversampling method. Nevertheless, as shown in the results, there was an improvement of up to 20% compared to algorithms trained on the original data across most tested datasets.

Regarding time and space complexity, WISEST adds modest computational overhead over vanilla SMOTE and all SMOTE-based approaches as it performs weighting and distance-threshold checks. In practice, these operations depend on the number of neighbors (*k*) and the dataset topology. However, most of the operations can be performed in constant time, while the highest cost (i.e., calculating the nearest neighbors) is the same for all SMOTE-like methods; that is, building an NN index (O(n · d)) and performing *m* k-neighbor queries (typical cost O(m · k · log n · d); worst-case O(m · n · d)), where *n* is the total number of samples, *d* is number of features, and *m* is the number of minority-class samples. The extra operations per returned neighbor are inexpensive vector arithmetic and a few scalar tests, so the asymptotic time complexity remains aligned with other NN-based samplers. Still, constant factors increase relative to plain SMOTE due to weighting, conditional branches, and occasional generation of multiple candidates per seed. Memory overhead is comparable too since, regarding the storage for the NN index plus O(nsyn · d) for synthetic points, WISEST may produce fewer or more synthetics than SMOTE depending on local weights, which temporarily affects peak memory usage. Compared to cleaning pipelines (e.g., SMOTE-ENN and Tomek+Links), WISEST can be faster overall because it often avoids an expensive second NN pass over the combined dataset. Now, compared to CGAN-based approaches (e.g., WCOM-KKNBR), WISEST is orders of magnitude cheaper in both runtime and memory since it does not need to train deep networks. Therefore, WISEST’s runtime is attractive for small-to-moderate *n* and moderate *d*.

WISEST provides a viable robust alternative to traditional oversampling methods, such as SMOTE and SMOTE-based approaches. However, as the results show, there is no universal solution for imbalanced datasets. All methods have their own potential contributions and limitations. Therefore, we believe it is necessary to run a pre-diagnostic on various parameters, such as IR, fraction border, and number of nearest neighbors (from minority to majority), sample distribution (i.e., local class overlap/noisy borders), and silhouette scores for minority clusters beforehand.

## 5. Conclusions

Imbalanced datasets significantly influence ML models. However, traditional oversampling methods, such as SMOTE, tend to generate unnecessary synthetic samples, including borderline samples, which can hinder detection. This paper introduces WISEST, a novel oversampling approach that uses a weighted location-aware strategy to increase sample counts near decision boundaries without generating many unsafe points within a threshold.

Through extensive experimentation, this paper showed that WISEST is effective on various datasets. Across the complete KEEL collection (low-imbalance, high-imbalance, and noisy/borderline variants), its primary strengths are consistent increases in recall and often net F1 gains. This was also the case on other multi-class datasets (i.e., IoT-23 and BoT–IoT) using different ML models. Thus, we can conclude that the WISEST conditional branching approach can help to address the dataset imbalance problem under the conditions described above.

Future directions for this work include a deep and formal sensitivity analysis and testing of dynamic thresholds and variables (e.g., θ,k) for multi-class datasets and their effects across different public real-world datasets, such as Credit Card Fraud (European cardholders), Mammography, and NSL-KDD (network intrusion; improved KDD99), among others. We also plan to include other ML models, such as SVM, CNN, and DNN, for evaluation. Finally, correlational and ablation analyses might shed further light on the influence of each part of the strategy (e.g., weighting and threshold distance).

## Figures and Tables

**Table 1:** Representative dataset groups from KEEL used for evaluation.

Dataset Group	Imbalance	Key Features
Ecoli subsets (e.g., ecoli-0-3-4_vs_5)	Medium–High	Borderline-prone; one-vs.-rest splits; minority pockets adjacent to the majority class, producing many local decision boundaries.
Yeast subsets (e.g., yeast-2_vs_4, yeast5)	Medium–High	Multimodal minority and majority pockets; some subsets are highly overlapping, while others are separable.
Glass subsets (e.g., glass-0-4_vs_5, glass4)	Low–Medium	Mix near-separable and borderline splits; small minority classes in some variants.
Abalone subsets (e.g., abalone-3_vs_11)	Medium–High	Sparse minority pockets and high heterogeneity in the majority; minority examples can be isolated or lie on tiny islands.
Shuttle pairs (e.g., shuttle-c0_vs_c4)	Low (sometimes trivial)	Large-scale with some perfectly separable splits; ideal as control cases to confirm oversamplers do not degrade already optimal classifiers and to test scalability.
Page-blocks/Vowel/Segment	Low–Medium	Well-structured feature space with clear local clusters.
Medical-type (Pima, Cleveland, Thyroid variants)	Low–Medium	Minority can be small and partially isolated; features can be noisy and clinically correlated.
KDD/Poker/large network subsets	Varies (often High)	Large mixed-type feature sets with some trivial and some degenerate pairs; includes extremely low-prevalence positives and categorical-heavy features.
Winequality subsets	High (often extreme)	Many splits have very low positive prevalence and degenerate metrics (zero precision/recall in some methods)
Noisy synthetic KEEL (03subcl, 04clover, paw02a with -BI)	Controlled (0–70% noise)	Designed to inject borderline/noisy examples; difficulty varies systematically with noise level: moderate noise tests robustness and targeted augmentation, high noise.

**Table 2:** Number of new synthetic minority samples produced per oversampling method (KEEL datasets with IR less than 9).

Dataset	SMOTE	Borderline-SMOTE	ADASYN	SMOTE + Tomek	SMOTEENN	Radius-SMOTE	WCOM-KKNBR	WISEST (Ours)
glass1	50	50	45	47	**22**	186	61	182
ecoli-0_vs_1	53	53	52	52	**48**	286	62	**48**
wisconsin	164	164	154	163	**145**	882	191	3786
pima	186	186	165	164	**22**	561	214	1101
iris0	40	**0**	**0**	40	40	196	40	91
glass0	59	59	60	57	**35**	191	56	163
yeast1	501	501	552	479	**259**	787	343	686
haberman	115	115	121	103	**44**	107	65	127
vehicle2	328	328	328	327	309	657	**174**	2397
vehicle1	330	330	320	324	221	398	**174**	538
vehicle3	338	338	344	330	222	337	**170**	497
glass-0-1-2-3_vs_4-5-6	90	90	90	90	82	155	**41**	406
vehicle0	358	358	359	358	344	643	**159**	2075
ecoli1	146	146	142	143	125	211	**62**	113
new-thyroid1	116	116	116	116	112	103	**28**	395
new-thyroid2	116	116	115	116	111	104	**28**	388
ecoli2	186	149	184	183	176	169	**42**	58
segment0	1320	1320	1319	1320	1318	1239	**263**	5749
glass6	125	100	125	125	118	90	**23**	307
yeast3	926	926	930	923	908	403	**130**	243
ecoli3	213	213	214	212	206	76	**28**	63
page-blocks0	3483	3483	3472	3469	3317	1446	**447**	5405

The lowest value in each row is highlighted in **bold**.

**Table 3:** Dataset characteristics of instances where WISEST created the fewest (above the mid line) and most synthetic samples (below the mid line).

Dataset	No. Minority	No. Majority	IR	Mean NN Majority Dist	Frac with Majority Neighbor k5
ecoli-0_vs_1	77	143	1.8571	0.3723	0.1429
ecoli1	77	259	3.3636	0.1726	0.7013
ecoli2	52	284	5.4615	0.1633	0.4615
ecoli3	35	301	8.6000	0.1198	0.8571
yeast3	163	1321	8.1043	0.0981	0.7239
wisconsin	239	444	1.8577	7.3735	0.2176
segment0	329	1979	6.0152	19.7524	0.0638
vehicle2	218	628	2.8807	39.6960	0.4862
vehicle0	199	647	3.2513	24.6629	0.4824
page-blocks0	559	4913	8.7889	1765.3490	0.5170
pima	268	500	1.8657	20.2823	0.8619

**Table 4:** KEEL dataset benchmark results for datasets with an IR less than or equal to 9.

Dataset	Metric	Original	SMOTE	Borderline- SMOTE	ADASYN	SMOTE+ Tomek	SMOTE ENN	Radius-SMOTE	WCOM KKNB	WISEST (Ours)
glass1	Accuracy	**0.8598**	0.8502	0.8504	0.8221	0.8410	0.7754	0.8269	0.8548	0.8032
	Precision	**0.8914**	0.8257	0.8041	0.7721	0.8115	0.6760	0.7662	0.8875	0.7188
	Recall	0.7100	0.7633	0.7900	0.7500	0.7500	0.7358	0.7633	0.6975	**0.8025**
	F1	0.7792	0.7858	**0.7908**	0.7511	0.7725	0.7014	0.7591	0.7765	0.7492
ecoli-0_vs_1	Accuracy	**0.9909**	**0.9909**	0.9773	0.9727	0.9864	**0.9909**	**0.9909**	**0.9909**	0.9864
	Precision	**1.0000**	**1.0000**	0.9624	0.9519	0.9882	**1.0000**	**1.0000**	**1.0000**	0.9882
	Recall	**0.9733**	**0.9733**	**0.9733**	**0.9733**	**0.9733**	**0.9733**	**0.9733**	**0.9733**	**0.9733**
	F1	**0.9862**	**0.9862**	0.9673	0.9616	0.9801	**0.9862**	**0.9862**	**0.9862**	0.9801
iris0	All	**1.0000**	**1.0000**	**1.0000**	-	**1.0000**	**1.0000**	**1.0000**	**1.0000**	**1.0000**
yeast1	Accuracy	0.7817	0.7642	0.7669	0.7554	0.7709	0.7291	0.7722	**0.7823**	0.7668
	Precision	0.6682	0.5906	0.5945	0.5719	0.5977	0.5233	0.6032	**0.6703**	0.5878
	Recall	0.4964	0.6247	0.6224	0.6363	0.6434	**0.7459**	0.6340	0.4942	0.6596
	F1	0.5673	0.6053	0.6072	0.6011	0.6190	0.6141	0.6171	0.5664	**0.6202**
haberman	Accuracy	0.6730	0.6600	0.6600	0.6632	0.6469	0.6501	0.6796	0.6731	**0.6894**
	Precision	0.3417	0.3561	0.3427	0.3715	0.3335	0.3829	0.3892	0.3435	**0.4083**
	Recall	0.2574	0.3449	0.3191	0.3941	0.3316	**0.5037**	0.3574	0.2463	0.3824
	F1	0.2925	0.3499	0.3296	0.3817	0.3316	**0.4321**	0.3715	0.2861	0.3935
glass-0-1-2- 3_vs_4-5-6	Accuracy	0.9392	0.9439	0.9485	0.9391	0.9439	0.9251	**0.9532**	**0.9532**	0.9483
	Precision	0.8859	0.8703	0.8808	0.8544	0.8703	0.8051	**0.8929**	0.8747	0.8488
	Recall	0.8618	0.9200	0.9200	0.9200	0.9200	0.9400	0.9200	0.9400	**0.9800**
	F1	0.8717	0.8901	0.8969	0.8813	0.8901	0.8615	0.9042	0.9052	**0.9067**
new-thyroid1	Accuracy	**0.9907**	0.9814	0.9721	0.9767	0.9814	**0.9907**	0.9814	0.9814	0.9860
	Precision	**1.0000**	0.9750	0.9306	0.9556	0.9750	0.9750	0.9750	0.9750	0.9306
	Recall	0.9429	0.9143	0.9143	0.9143	0.9143	0.9714	0.9143	0.9143	**1.0000**
	F1	0.9692	0.9379	0.9129	0.9263	0.9379	**0.9713**	0.9379	0.9379	0.9617
new-thyroid2	Accuracy	**0.9860**	0.9767	0.9814	0.9767	0.9767	0.9814	0.9814	0.9814	**0.9860**
	Precision	**0.9750**	**0.9750**	**0.9750**	0.9556	**0.9750**	**0.9750**	**0.9750**	**0.9750**	0.9500
	Recall	0.9429	0.8857	0.9143	0.9143	0.8857	0.9143	0.9143	0.9143	**0.9714**
	F1	0.9533	0.9167	0.9379	0.9263	0.9167	0.9379	0.9379	0.9405	**0.9579**
glass6	Accuracy	0.9627	**0.9813**	0.9720	0.9766	**0.9813**	0.9673	0.9673	0.9719	0.9720
	Precision	**0.9667**	**0.9667**	**0.9667**	0.9381	**0.9667**	0.9333	**0.9667**	0.9429	**0.9667**
	Recall	0.7667	**0.9000**	0.8333	**0.9000**	**0.9000**	0.8333	0.8000	0.8667	0.8333
	F1	0.8436	**0.9273**	0.8873	0.9119	**0.9273**	0.8721	0.8655	0.8939	0.8873

The highest value, including ties in each row, is highlighted in **bold**. Cells with no value (‘-’) denote instances where the oversampling algorithm could not be calculated.

**Table 5:** Number of new synthetic minority samples per oversampling method for KEEL datasets. Top 10 WISEST lowest above the mid line and top 10 highest below.

Dataset	SMOTE	Borderline- SMOTE	ADASYN	SMOTE +Tomek	SMOTEENN	Radius- SMOTE	WCOM KKNBR	WISEST (Ours)
winequality-red-3_vs_5	537	537	538	532	488	1	8	1
winequality-white-9_vs_4	0	0	0	0	0	1	0	1
abalone19	3288	3288	3285	3284	3245	3	26	3
poker-8_vs_6	1154	1154	1154	1154	1154	3	14	3
winequality-red-8_vs_6-7	655	524	657	643	558	1	14	3
winequality-white-3-9_vs_5	1146	1146	1142	1121	949	3	20	3
abalone-3_vs_11	378	302	378	378	378	52	12	4
lymphography-normal-fibrosis	0	0	0	0	0	4	4	4
winequality-red-8_vs_6	496	496	495	485	422	2	14	4
abalone-19_vs_10-11-12-13	1246	1246	1246	1241	1193	5	26	5
ecoli-0-1_vs_2-3-5	157	157	156	156	148	64	19	260
ecoli-0-1-4-6_vs_5	192	192	192	192	188	61	16	269
dermatology-6	254	203	254	254	254	73	16	333
kddcup-land_vs_portsweep	815	0	0	815	815	81	17	378
vowel0	646	646	646	646	646	290	72	540
kddcup-buffer_overflow_vs_back	1738	695	1739	1738	1738	115	24	563
shuttle-2_vs_5	2574	2574	2575	2574	2574	191	39	923
kddcup-guess_passwd_vs_satan	1229	0	0	1229	1229	212	42	1060
kr-vs-k-one_vs_fifteen	1670	1670	1671	1670	1670	304	62	1428
kr-vs-k-zero-one_vs_draw	2153	2153	2152	2152	2149	358	84	1542
shuttle-c0-vs-c4	1266	760	1266	1266	1264	486	98	2410

**Table 6:** Dataset characteristics of instances where WISEST created the fewest (above the mid line) and most synthetic samples (below the mid line) for datasets with IR greater than 9.

Dataset	No. Minority	No. Majority	IR	Mean NN Majority Dist	Frac with Majority Neighbor k5
abalone19	32	4142	129.4	0.05	1
glass5	9	205	22.8	1.5	1
poker-9_vs_7	8	236	29.5	4.5	1
winequality-red-4	53	1546	29.2	2.0	1
ecoli-0-1_vs_2-3-5	24	220	9.2	33.9	0.46
dermatology-6	20	338	16.9	5.5	0.15
kr-vs-k-one_vs_fifteen	78	2166	27.7	2.7	0.05
shuttle-c0_vs_c4	123	1706	13.9	139.1	0.02

**Table 7:** KEEL benchmark results (accuracy, precision, recall, and F1) for all datasets whose IR is greater than 9. Selected results where WISEST achieved the highest value in at least one of the metrics (including ties).

Dataset	Metric	Original	SMOTE	Borderline- SMOTE	ADASYN	SMOTE +Tomek	SMOTE ENN	Radius-SMOTE	WCOM KKNB	WISEST (Ours)
vowel0	Accuracy	0.9949	**0.9960**	**0.9960**	0.9949	**0.9960**	**0.9960**	0.9949	0.9919	0.9959
	Precision	**0.9895**	0.9789	0.9789	0.9695	0.9789	0.9789	0.9784	**0.9895**	0.9684
	Recall	0.9556	0.9778	0.9778	0.9778	0.9778	0.9778	0.9667	0.9222	**0.9889**
	F1	0.9714	0.9778	0.9778	0.9726	0.9778	0.9778	0.9721	0.9529	**0.9781**
shuttle-c0-vs-c4	All	**1.0000**	**1.0000**	**1.0000**	**1.0000**	**1.0000**	**1.0000**	**1.0000**	**1.0000**	**1.0000**
page-blocks-1- 3_vs_4	Accuracy	0.9916	0.9958	0.9979	0.9979	0.9958	0.9958	**1.0000**	0.9916	0.9958
	Precision	0.9667	**1.0000**	**1.0000**	**1.0000**	**1.0000**	**1.0000**	**1.0000**	0.9667	0.9381
	Recall	0.9000	0.9333	0.9667	0.9667	0.9333	0.9333	**1.0000**	0.9000	**1.0000**
	F1	0.9236	0.9636	0.9818	0.9818	0.9636	0.9636	**1.0000**	0.9236	0.9664
glass-0-1- 6_vs_5	Accuracy	0.9782	0.9835	0.9835	0.9835	0.9835	**0.9889**	0.9781	0.9836	0.9782
	Precision	0.8000	0.8000	0.8000	0.8000	0.8000	0.8000	0.8000	**0.9000**	0.8000
	Recall	**0.8000**	0.7000	0.7000	0.7000	0.7000	**0.8000**	0.6000	**0.8000**	**0.8000**
	F1	0.7667	0.7333	0.7333	0.7333	0.7333	**0.8000**	0.6667	**0.8000**	0.7667
shuttle-c2- vs-c4	Accuracy	0.9923	-	-	-	-	-	0.9923	0.9923	**1.0000**
	Precision	**1.0000**	-	-	-	-	-	**1.0000**	**1.0000**	**1.0000**
	Recall	0.9000	-	-	-	-	-	0.9000	0.9000	**1.0000**
	F1	0.9333	-	-	-	-	-	0.9333	0.9333	**1.0000**
yeast-2_vs_8	Accuracy	0.9751	0.9606	0.9564	0.9398	0.9585	0.9357	0.9751	**0.9792**	0.9751
	Precision	**0.9500**	0.5676	0.4667	0.3000	0.5750	0.3294	**0.9500**	**0.9500**	**0.9500**
	Recall	0.4500	0.5000	0.1500	0.3000	0.5000	**0.5500**	0.4500	**0.5500**	0.4500
	F1	0.5614	0.4731	0.2171	0.2889	0.4638	0.3884	0.5614	**0.6433**	0.5614
yeast5	Accuracy	0.9811	0.9838	0.9825	0.9825	0.9838	0.9805	**0.9852**	0.9818	**0.9852**
	Precision	**0.8250**	0.7060	0.6769	0.6839	0.7060	0.6315	0.7739	0.8100	0.7589
	Recall	0.4917	0.8111	0.8083	0.7861	0.8111	**0.8806**	0.7222	0.5611	0.7444
	F1	0.5631	0.7400	0.7130	0.7094	0.7400	0.7254	0.7369	0.6212	**0.7406**
ecoli-0-1- 4-7_vs_5-6	Accuracy	0.9759	0.9668	0.9698	0.9668	0.9698	0.9638	0.9759	0.9759	**0.9820**
	Precision	**0.9500**	0.7700	0.8433	0.7944	0.8033	0.7643	**0.9500**	**0.9500**	0.9333
	Recall	0.7200	0.8000	0.7600	**0.8400**	0.8000	0.8000	0.7200	0.7200	**0.8400**
	F1	0.8111	0.7806	0.7888	0.7994	0.7988	0.7717	0.8111	0.8111	**0.8732**
dermatology-6	All	**1.0000**	**1.0000**	**1.0000**	**1.0000**	**1.0000**	**1.0000**	**1.0000**	**1.0000**	**1.0000**
winequality- red-8_vs_6-7	Accuracy	**0.9813**	0.9602	0.9801	0.9602	0.9614	0.9216	**0.9813**	**0.9813**	**0.9813**
	Precision	**0.4000**	0.1333	**0.4000**	0.1333	0.1467	0.1103	**0.4000**	**0.4000**	**0.4000**
	Recall	0.1000	0.2000	0.1000	0.2000	0.2000	**0.3833**	0.1000	0.1000	0.1000
	F1	0.1600	0.1600	0.1600	0.1600	0.1689	**0.1700**	0.1600	0.1600	0.1600

The highest value, including ties in each row, is highlighted in **bold**. Cells with no value (‘-’) denote instances where the oversampling algorithm could not be calculated.

**Table 8:** KEEL benchmark results (accuracy, precision, recall, and F1) for all noisy/borderline datasets. Selected results where WISEST achieved the highest value in at least one of the metrics (including ties).

Dataset	Metric	Original	SMOTE	Borderline- SMOTE	ADASYN	SMOTE +Tomek	SMOTE ENN	Radius-SMOTE	WCOM KKNB	WISEST (Ours)
03subcl5-600-5-0-BI	Accuracy	0.9483	0.9467	0.9450	0.9483	0.9450	0.9233	0.9400	**0.9533**	0.9483
	Precision	0.8378	0.8235	0.8131	0.8181	0.8176	0.7263	0.7981	**0.8771**	0.7810
	Recall	0.8600	0.8700	0.8900	0.9000	0.8700	0.8800	0.8700	0.8400	**0.9700**
	F1	0.8457	0.8430	0.8454	0.8542	0.8403	0.7940	0.8292	0.8551	**0.8641**
03subcl5-800-7-0-BI	Accuracy	0.9538	0.9525	0.9538	**0.9575**	0.9550	0.9462	0.9550	0.9563	0.9550
	Precision	0.8212	0.7744	0.7896	0.7963	0.7878	0.7274	0.7920	**0.8560**	0.7662
	Recall	0.8100	0.8800	0.8700	0.8900	0.8800	0.9100	0.8700	0.7800	**0.9300**
	F1	0.8149	0.8224	0.8235	**0.8391**	0.8297	0.8067	0.8283	0.8155	0.8387
03subcl5-800-7-30-BI	Accuracy	**0.9113**	0.8613	0.8588	0.8575	0.8575	0.8263	0.9025	0.9088	0.9025
	Precision	**0.6994**	0.4647	0.4618	0.4707	0.4552	0.4012	0.6079	0.6919	0.6117
	Recall	0.5400	0.6900	0.6900	**0.7400**	0.6800	**0.7400**	0.6300	0.5200	0.6600
	F1	0.5990	0.5537	0.5514	0.5707	0.5434	0.5185	0.6149	0.5873	**0.6324**
04clover5z-600-5-0-BI	Accuracy	0.9200	0.9150	0.9200	0.9083	0.9200	0.8967	0.9150	**0.9350**	0.9150
	Precision	0.7802	0.7181	0.7166	0.6819	0.7235	0.6446	0.7256	**0.8438**	0.6931
	Recall	0.7400	0.8300	0.8700	0.8700	0.8700	0.8900	0.8100	0.7600	**0.9000**
	F1	0.7559	0.7683	0.7844	0.7625	0.7867	0.7451	0.7623	**0.7969**	0.7816
04clover5z-600-5-30-BI	Accuracy	**0.8717**	0.8667	0.8600	0.8600	0.8600	0.8283	0.8683	0.8700	0.8700
	Precision	**0.6573**	0.5875	0.5672	0.5615	0.5690	0.5011	0.6067	0.6446	0.5955
	Recall	0.4900	0.7200	0.7300	0.7500	0.7100	**0.8000**	0.6300	0.5100	0.7100
	F1	0.5569	0.6438	0.6365	0.6406	0.6294	0.6113	0.6113	0.5642	**0.6451**
paw02a-800-7-0-BI	Accuracy	0.9712	0.9688	**0.9737**	0.9700	0.9688	0.9650	0.9700	0.9713	0.9675
	Precision	0.8884	0.8417	0.8793	0.8449	0.8417	0.8072	0.8592	**0.8920**	0.8285
	Recall	0.8900	0.9300	0.9200	0.9400	0.9300	**0.9500**	0.9200	0.8900	**0.9500**
	F1	0.8864	0.8820	**0.8981**	0.8879	0.8820	0.8717	0.8856	0.8870	0.8816

For each metric, the highest value is shown in **bold**.

**Table 9:** Benchmark results by dataset, metric, and classifier (original vs. WISEST).

Dataset	Metric	KNN		Random Forest		LightGBM	
		**Original**	**WISEST (Ours)**	**Original**	**WISEST (Ours)**	**Original**	**WISEST (Ours)**
IoT-23	Accuracy	0.655	0.459	0.681	0.590	0.665	0.642
	Precision	0.505	**0.765**	0.765	**0.774**	0.759	**0.798**
	Recall	0.537	**0.649**	0.532	**0.593**	0.504	**0.579**
	F1	0.484	**0.589**	0.548	**0.586**	0.497	**0.575**
BoT–IoT	Accuracy	0.911	0.910	0.916	0.913	0.914	0.913
	Precision	0.338	**0.362**	0.390	**0.414**	0.376	**0.541**
	Recall	0.345	**0.360**	0.346	**0.347**	0.535	0.377
	F1	0.333	**0.358**	0.335	**0.340**	0.389	**0.391**
Air and Pollution	Accuracy	0.918	0.897	0.960	0.949	0.959	0.949
	Precision	0.904	0.857	0.943	0.925	0.950	0.925
	Recall	0.874	0.855	0.938	0.925	0.935	0.926
	F1	0.886	0.850	0.941	0.926	0.942	0.925

Bold entries indicate WISEST is higher than original for that classifier/metric.

## Data Availability

The original datasets presented in the study are openly available from the KEEL (Knowledge Extraction based on Evolutionary Learning) repository at https://sci2s.ugr.es/keel/ (accessed on 27 November 2025) or Kaggle at https://www.kaggle.com/ (accessed on 27 November 2025).

## Appendix A

**Table A1:** Full KEEL dataset benchmark results for datasets with an IR less than or equal to 9.

Dataset	Metric	Original	SMOTE	Borderline- SMOTE	ADASYN	SMOTE +Tomek	SMOTEENN	Radius-SMOTE	WCOM-KKNBR	WISEST
glass1	Accuracy	0.8598	0.8502	0.8504	0.8221	0.8410	0.7754	0.8269	0.8548	0.8032
	Precision	0.8914	0.8257	0.8041	0.7721	0.8115	0.6760	0.7662	0.8875	0.7188
	Recall	0.7100	0.7633	0.7900	0.7500	0.7500	0.7358	0.7633	0.6975	0.8025
	F1	0.7792	0.7858	0.7908	0.7511	0.7725	0.7014	0.7591	0.7765	0.7492
ecoli-0_vs_1	Accuracy	0.9909	0.9909	0.9773	0.9727	0.9864	0.9909	0.9909	0.9909	0.9864
	Precision	1.0000	1.0000	0.9624	0.9519	0.9882	1.0000	1.0000	1.0000	0.9882
	Recall	0.9733	0.9733	0.9733	0.9733	0.9733	0.9733	0.9733	0.9733	0.9733
	F1	0.9862	0.9862	0.9673	0.9616	0.9801	0.9862	0.9862	0.9862	0.9801
wisconsin	Accuracy	0.9736	0.9736	0.9678	0.9707	0.9751	0.9736	0.9766	0.9678	0.9737
	Precision	0.9512	0.9445	0.9435	0.9436	0.9446	0.9446	0.9482	0.9370	0.9444
	Recall	0.9748	0.9832	0.9664	0.9747	0.9874	0.9832	0.9873	0.9748	0.9832
	F1	0.9627	0.9632	0.9546	0.9587	0.9654	0.9632	0.9672	0.9552	0.9633
pima	Accuracy	0.7630	0.7473	0.7434	0.7604	0.7604	0.7304	0.7473	0.7643	0.7421
	Precision	0.6879	0.6317	0.6196	0.6443	0.6496	0.5855	0.6211	0.6921	0.6073
	Recall	0.5897	0.6716	0.7015	0.7088	0.6865	0.8133	0.7239	0.5857	0.7574
	F1	0.6342	0.6501	0.6570	0.6745	0.6666	0.6792	0.6676	0.6343	0.6729
iris0	All	1.0000	1.0000	1.0000		1.0000	1.0000	1.0000	1.0000	1.0000
glass0	Accuracy	0.8693	0.8600	0.8599	0.8506	0.8553	0.8179	0.8600	0.8739	0.8553
	Precision	0.8125	0.7589	0.7550	0.7245	0.7595	0.6635	0.7515	0.8243	0.7360
	Recall	0.8000	0.8429	0.8429	0.8857	0.8143	0.9000	0.8571	0.8000	0.8714
	F1	0.8000	0.7981	0.7957	0.7962	0.7842	0.7628	0.8003	0.8065	0.7978
yeast1	Accuracy	0.7817	0.7642	0.7669	0.7554	0.7709	0.7291	0.7722	0.7823	0.7668
	Precision	0.6682	0.5906	0.5945	0.5719	0.5977	0.5233	0.6032	0.6703	0.5878
	Recall	0.4964	0.6247	0.6224	0.6363	0.6434	0.7459	0.6340	0.4942	0.6596
	F1	0.5673	0.6053	0.6072	0.6011	0.6190	0.6141	0.6171	0.5664	0.6202
haberman	Accuracy	0.6730	0.6600	0.6600	0.6632	0.6469	0.6501	0.6796	0.6731	0.6894
	Precision	0.3417	0.3561	0.3427	0.3715	0.3335	0.3829	0.3892	0.3435	0.4083
	Recall	0.2574	0.3449	0.3191	0.3941	0.3316	0.5037	0.3574	0.2463	0.3824
	F1	0.2925	0.3499	0.3296	0.3817	0.3316	0.4321	0.3715	0.2861	0.3935
vehicle2	Accuracy	0.9870	0.9846	0.9834	0.9858	0.9846	0.9728	0.9858	0.9858	0.9787
	Precision	0.9817	0.9734	0.9685	0.9728	0.9734	0.9204	0.9693	0.9816	0.9401
	Recall	0.9678	0.9678	0.9678	0.9723	0.9678	0.9815	0.9771	0.9632	0.9814
	F1	0.9744	0.9701	0.9679	0.9724	0.9701	0.9492	0.9727	0.9721	0.9597
vehicle1	Accuracy	0.7932	0.7884	0.7860	0.7860	0.7896	0.7471	0.7860	0.7943	0.7766
	Precision	0.6330	0.5800	0.5756	0.5809	0.5834	0.5061	0.5792	0.6367	0.5567
	Recall	0.4748	0.6451	0.6357	0.6124	0.6311	0.8200	0.6171	0.4747	0.6448
	F1	0.5399	0.6085	0.6030	0.5923	0.6044	0.6247	0.5933	0.5428	0.5955
vehicle3	Accuracy	0.7896	0.7671	0.7825	0.7719	0.7766	0.7328	0.7766	0.7932	0.7707
	Precision	0.6218	0.5348	0.5645	0.5437	0.5518	0.4832	0.5563	0.6297	0.5405
	Recall	0.4062	0.5948	0.6226	0.5946	0.5998	0.7973	0.5571	0.4159	0.6092
	F1	0.4885	0.5616	0.5909	0.5666	0.5729	0.6006	0.5552	0.4975	0.5714
glass-0-1-2- 3_vs_4-5-6	Accuracy	0.9392	0.9439	0.9485	0.9391	0.9439	0.9251	0.9532	0.9532	0.9483
	Precision	0.8859	0.8703	0.8808	0.8544	0.8703	0.8051	0.8929	0.8747	0.8488
	Recall	0.8618	0.9200	0.9200	0.9200	0.9200	0.9400	0.9200	0.9400	0.9800
	F1	0.8717	0.8901	0.8969	0.8813	0.8901	0.8615	0.9042	0.9052	0.9067
vehicle0	Accuracy	0.9716	0.9681	0.9752	0.9704	0.9669	0.9350	0.9633	0.9693	0.9551
	Precision	0.9447	0.9100	0.9330	0.9112	0.9095	0.7937	0.9050	0.9398	0.8580
	Recall	0.9345	0.9596	0.9646	0.9696	0.9546	0.9800	0.9445	0.9294	0.9696
	F1	0.9393	0.9339	0.9482	0.9392	0.9313	0.8769	0.9237	0.9342	0.9102
ecoli1	Accuracy	0.9108	0.9168	0.8989	0.8931	0.9168	0.8751	0.9079	0.9048	0.9049
	Precision	0.8231	0.7994	0.7488	0.7197	0.7925	0.6671	0.7922	0.8117	0.7717
	Recall	0.8033	0.8825	0.8817	0.9217	0.8950	0.9342	0.8417	0.7900	0.8683
	F1	0.7942	0.8314	0.8015	0.8026	0.8333	0.7750	0.8053	0.7856	0.8052
new- thyroid1	Accuracy	0.9907	0.9814	0.9721	0.9767	0.9814	0.9907	0.9814	0.9814	0.9860
	Precision	1.0000	0.9750	0.9306	0.9556	0.9750	0.9750	0.9750	0.9750	0.9306
	Recall	0.9429	0.9143	0.9143	0.9143	0.9143	0.9714	0.9143	0.9143	1.0000
	F1	0.9692	0.9379	0.9129	0.9263	0.9379	0.9713	0.9379	0.9379	0.9617
new- thyroid2	Accuracy	0.9860	0.9767	0.9814	0.9767	0.9767	0.9814	0.9814	0.9814	0.9860
	Precision	0.9750	0.9750	0.9750	0.9556	0.9750	0.9750	0.9750	0.9750	0.9500
	Recall	0.9429	0.8857	0.9143	0.9143	0.8857	0.9143	0.9143	0.9143	0.9714
	F1	0.9533	0.9167	0.9379	0.9263	0.9167	0.9379	0.9379	0.9405	0.9579
ecoli2	Accuracy	0.9464	0.9494	0.9554	0.9286	0.9494	0.9583	0.9583	0.9523	0.9494
	Precision	0.8967	0.8689	0.9044	0.7485	0.8495	0.8521	0.9063	0.9181	0.8452
	Recall	0.7327	0.7909	0.7891	0.8091	0.8091	0.8873	0.8091	0.7527	0.8273
	F1	0.8008	0.8182	0.8364	0.7742	0.8233	0.8674	0.8469	0.8183	0.8252
segment0	Accuracy	0.9974	0.9970	0.9970	0.9974	0.9965	0.9957	0.9965	0.9974	0.9957
	Precision	0.9969	0.9909	0.9909	0.9910	0.9908	0.9849	0.9881	0.9969	0.9821
	Recall	0.9848	0.9878	0.9878	0.9909	0.9848	0.9848	0.9878	0.9848	0.9878
	F1	0.9908	0.9893	0.9893	0.9909	0.9878	0.9848	0.9879	0.9908	0.9849
glass6	Accuracy	0.9627	0.9813	0.9720	0.9766	0.9813	0.9673	0.9673	0.9719	0.9720
	Precision	0.9667	0.9667	0.9667	0.9381	0.9667	0.9333	0.9667	0.9429	0.9667
	Recall	0.7667	0.9000	0.8333	0.9000	0.9000	0.8333	0.8000	0.8667	0.8333
	F1	0.8436	0.9273	0.8873	0.9119	0.9273	0.8721	0.8655	0.8939	0.8873
yeast3	Accuracy	0.9488	0.9508	0.9474	0.9515	0.9488	0.9421	0.9481	0.9481	0.9488
	Precision	0.8173	0.7572	0.7347	0.7546	0.7497	0.6853	0.7814	0.7988	0.7675
	Recall	0.6987	0.8277	0.8341	0.8402	0.8218	0.9019	0.7477	0.7235	0.7786
	F1	0.7482	0.7881	0.7783	0.7928	0.7799	0.7765	0.7600	0.7541	0.7704
ecoli3	Accuracy	0.9345	0.9137	0.9047	0.8958	0.9137	0.8839	0.9227	0.9226	0.9167
	Precision	0.8000	0.5675	0.5336	0.5050	0.5675	0.4703	0.6694	0.7329	0.6152
	Recall	0.5143	0.7143	0.5714	0.6857	0.7143	0.8571	0.6000	0.4571	0.6000
	F1	0.6151	0.6259	0.5462	0.5707	0.6259	0.6062	0.6192	0.5408	0.6005
page-blocks0	Accuracy	0.9762	0.9720	0.9706	0.9686	0.9715	0.9607	0.9761	0.9764	0.9746
	Precision	0.8893	0.8247	0.8140	0.8001	0.8211	0.7384	0.8809	0.8883	0.8597
	Recall	0.8766	0.9231	0.9231	0.9249	0.9231	0.9535	0.8855	0.8801	0.8981
	F1	0.8828	0.8708	0.8649	0.8574	0.8687	0.8321	0.8830	0.8840	0.8784

## Appendix B

**Table A2:** Number of new synthetic minority samples per oversampling method for KEEL datasets with an IR greater than 9.

Dataset	SMOTE	Borderline- SMOTE	ADASYN	SMOTE +Tomek	SMOTEENN	Radius-SMOTE	WCOM-KKNBR	WISEST
yeast-2_vs_4	330	330	333	329	322	125	41	61
yeast-0-5-6-7-9_vs_4	341	341	339	338	321	72	41	65
vowel0	646	646	646	646	646	290	72	540
glass-0-1-6_vs_2	126	126	126	125	115	14	14	14
shuttle-c0-vs-c4	1266	760	1266	1266	1264	486	98	2410
yeast-1_vs_7	319	319	321	316	301	21	24	21
glass4	150	150	150	150	150	25	10	37
ecoli4	237	237	236	236	235	59	16	22
page-blocks-1-3_vs_4	333	333	333	333	330	70	22	186
abalone9-18	518	518	519	511	467	28	34	28
glass-0-1-6_vs_5	133	133	132	133	133	12	7	12
shuttle-c2-vs-c4	0	0	0	0	0	8	4	8
yeast-1-4-5-8_vs_7	506	506	504	505	489	11	24	11
glass5	157	157	157	157	157	11	7	11
yeast-2_vs_8	354	283	353	351	338	44	16	10
yeast4	1106	1106	1110	1103	1086	49	41	47
yeast-1-2-8-9_vs_7	710	710	710	708	689	13	24	13
yeast5	1117	1117	1115	1117	1115	101	35	87
yeast6	1131	1131	1131	1129	1114	66	28	55
abalone19	3288	3288	3285	3284	3245	3	26	3
cleveland-0_vs_4	118	118	118	113	99	6	10	6
ecoli-0-1_vs_2-3-5	157	157	156	156	148	64	19	260
ecoli-0-1_vs_5	160	160	160	159	156	60	16	256
ecoli-0-1-4-6_vs_5	192	192	192	192	188	61	16	269
ecoli-0-1-4-7_vs_2-3-5-6	222	222	222	221	212	67	23	167
ecoli-0-1-4-7_vs_5-6	226	226	225	225	222	64	20	164
ecoli-0-2-3-4_vs_5	130	130	129	129	126	61	16	253
ecoli-0-2-6-7_vs_3-5	144	144	143	143	138	51	18	131
ecoli-0-3-4_vs_5	128	128	128	127	123	61	16	253
ecoli-0-3-4-7_vs_5-6	166	166	164	164	159	64	20	172
ecoli-0-4-6_vs_5	130	130	130	130	127	61	16	257
ecoli-0-6-7_vs_3-5	142	142	144	142	137	51	18	139
ecoli-0-6-7_vs_5	144	144	144	144	142	49	16	141
glass-0-1-4-6_vs_2	137	137	137	136	128	16	14	16
glass-0-1-5_vs_2	110	110	110	109	101	15	14	15
glass-0-4_vs_5	59	59	58	59	59	17	7	33
glass-0-6_vs_5	72	72	73	72	71	13	7	13
led7digit-0-2-4-5-6-7-8-9_vs_1	295	295	295	295	105	26	30	128
yeast-0-2-5-6_vs_3-7-8-9	645	645	648	638	600	189	79	144
yeast-0-2-5-7-9_vs_3-6-8	645	645	647	643	630	291	79	112
yeast-0-3-5-9_vs_7-8	325	325	324	322	301	68	40	55
abalone-3_vs_11	378	302	378	378	378	52	12	4
abalone-17_vs_7-8-9-10	1778	1778	1776	1776	1758	37	46	37
abalone-19_vs_10-11-12-13	1246	1246	1246	1241	1193	5	26	5
abalone-20_vs_8-9-10	1491	1491	1492	1489	1478	9	21	9
abalone-21_vs_8	442	442	442	442	436	12	11	12
car-good	1272	1272	1271	1272	1130	41	55	69
car-vgood	1278	1278	1281	1278	1117	49	52	78
dermatology-6	254	203	254	254	254	73	16	333
flare-F	784	784	789	783	680	16	34	38
kddcup-buffer_overflow_vs_back	1738	695	1739	1738	1738	115	24	563
kddcup-guess_passwd_vs_satan	1229	0	0	1229	1229	212	42	1060
kddcup-land_vs_portsweep	815	0	0	815	815	81	17	378
kr-vs-k-one_vs_fifteen	1670	1670	1671	1670	1670	304	62	1428
kr-vs-k-zero-one_vs_draw	2153	2153	2152	2152	2149	358	84	1542
lymphography-normal-fibrosis	0	0	0	0	0	4	4	4
poker-8_vs_6	1154	1154	1154	1154	1154	3	14	3
poker-8-9_vs_5	1620	1620	1618	1620	1615	7	20	7
poker-9_vs_7	182	182	182	182	181	5	6	5
shuttle-2_vs_5	2574	2574	2575	2574	2574	191	39	923
shuttle-6_vs_2-3	168	168	134	168	168	38	8	182
winequality-red-3_vs_5	537	537	538	532	488	1	8	1
winequality-red-4	1194	1194	1183	1179	1062	8	42	8
winequality-red-8_vs_6-7	655	524	657	643	558	1	14	3
winequality-red-8_vs_6	496	496	495	485	422	2	14	4
winequality-white-3_vs_7	688	688	688	679	598	8	16	8
winequality-white-3-9_vs_5	1146	1146	1142	1121	949	3	20	3
winequality-white-9_vs_4	0	0	0	0	0	1	0	1
zoo-3	0	0	0	0	0	4	0	4

## Appendix C

**Table A3:** Full KEEL benchmark results (accuracy, precision, recall, and F1) for datasets with IR greater than 9.

Dataset	Metric	Original	SMOTE	Borderline- SMOTE	ADASYN	SMOTE +Tomek	SMOTE ENN	Radius-SMOTE	WCOM KKNB	WISEST (Ours)
yeast- 2_vs_4	Accuracy	0.9631	0.9573	0.9495	0.9495	0.9553	0.9417	0.9592	0.9592	0.9592
	Precision	0.9010	0.7818	0.7733	0.7306	0.7697	0.6547	0.8373	0.8639	0.8465
	Recall	0.7273	0.8055	0.7255	0.8273	0.8055	0.9018	0.7473	0.7273	0.7473
	F1	0.7948	0.7921	0.7391	0.7698	0.7851	0.7541	0.7828	0.7735	0.7842
yeast-0-5- 6-7-9_vs_4	Accuracy	0.9185	0.9223	0.9090	0.9185	0.9166	0.8882	0.9242	0.9186	0.9166
	Precision	0.7500	0.5930	0.5231	0.5676	0.5642	0.4611	0.6640	0.7533	0.5906
	Recall	0.2745	0.6455	0.5255	0.6436	0.6255	0.7655	0.4691	0.2945	0.4673
	F1	0.3981	0.6171	0.5206	0.6007	0.5926	0.5710	0.5448	0.4100	0.5135
vowel0	Accuracy	0.9949	0.9960	0.9960	0.9949	0.9960	0.9960	0.9949	0.9919	0.9959
	Precision	0.9895	0.9789	0.9789	0.9695	0.9789	0.9789	0.9784	0.9895	0.9684
	Recall	0.9556	0.9778	0.9778	0.9778	0.9778	0.9778	0.9667	0.9222	0.9889
	F1	0.9714	0.9778	0.9778	0.9726	0.9778	0.9778	0.9721	0.9529	0.9781
glass-0-1- 6_vs_2	Accuracy	0.9116	0.9012	0.8958	0.8853	0.8960	0.8228	0.9065	0.9116	0.9012
	Precision	0.0000	0.5333	0.4333	0.4571	0.5000	0.2535	0.0667	0.0000	0.0667
	Recall	0.0000	0.3500	0.2333	0.3667	0.3500	0.5167	0.0500	0.0000	0.0500
	F1	0.0000	0.3867	0.2838	0.3505	0.3733	0.3369	0.0571	0.0000	0.0571
shuttle-c0-vs-c4	All	1.0000	1.0000	1.0000	1.0000	1.0000	1.0000	1.0000	1.0000	1.0000
yeast- 1_vs_7	Accuracy	0.9434	0.9151	0.9216	0.9064	0.9107	0.8474	0.9390	0.9412	0.9346
	Precision	0.4500	0.3424	0.3500	0.2803	0.2967	0.2047	0.4500	0.4333	0.3300
	Recall	0.2667	0.4000	0.3667	0.3333	0.3667	0.4667	0.3000	0.2333	0.2667
	F1	0.3333	0.3630	0.3571	0.2993	0.3242	0.2818	0.3518	0.3022	0.2927
glass4	Accuracy	0.9626	0.9671	0.9813	0.9671	0.9671	0.9529	0.9719	0.9626	0.9672
	Precision	0.7000	0.6833	0.8000	0.6833	0.6833	0.6267	0.8000	0.7000	0.6500
	Recall	0.5000	0.8333	0.9333	0.8333	0.8333	0.8333	0.7667	0.5000	0.7333
	F1	0.5600	0.7448	0.8533	0.7448	0.7448	0.7005	0.7533	0.5600	0.6648
ecoli4	Accuracy	0.9762	0.9703	0.9614	0.9644	0.9703	0.9674	0.9792	0.9762	0.9792
	Precision	0.9333	0.8000	0.6967	0.7300	0.8000	0.7633	0.8833	0.9600	0.8667
	Recall	0.6500	0.6500	0.6500	0.6500	0.6500	0.7000	0.7500	0.6500	0.7500
	F1	0.7524	0.7095	0.6675	0.6770	0.7095	0.7246	0.8071	0.7492	0.8000
page-blocks- 1-3_vs_4	Accuracy	0.9916	0.9958	0.9979	0.9979	0.9958	0.9958	1.0000	0.9916	0.9958
	Precision	0.9667	1.0000	1.0000	1.0000	1.0000	1.0000	1.0000	0.9667	0.9381
	Recall	0.9000	0.9333	0.9667	0.9667	0.9333	0.9333	1.0000	0.9000	1.0000
	F1	0.9236	0.9636	0.9818	0.9818	0.9636	0.9636	1.0000	0.9236	0.9664
abalone9-18	Accuracy	0.9480	0.9152	0.9289	0.9138	0.9193	0.8769	0.9494	0.9508	0.9467
	Precision	0.5000	0.3820	0.4233	0.3782	0.4320	0.2476	0.6767	0.7000	0.5333
	Recall	0.1222	0.5056	0.4111	0.4806	0.5278	0.5278	0.2444	0.1694	0.2444
	F1	0.1964	0.4092	0.3949	0.3987	0.4369	0.3251	0.3364	0.2655	0.3216
glass-0-1- 6_vs_5	Accuracy	0.9782	0.9835	0.9835	0.9835	0.9835	0.9889	0.9781	0.9836	0.9782
	Precision	0.8000	0.8000	0.8000	0.8000	0.8000	0.8000	0.8000	0.9000	0.8000
	Recall	0.8000	0.7000	0.7000	0.7000	0.7000	0.8000	0.6000	0.8000	0.8000
	F1	0.7667	0.7333	0.7333	0.7333	0.7333	0.8000	0.6667	0.8000	0.7667
shuttle-c2- vs-c4	Accuracy	0.9923	-	-	-	-	-	0.9923	0.9923	1.0000
	Precision	1.0000	-	-	-	-	-	1.0000	1.0000	1.0000
	Recall	0.9000	-	-	-	-	-	0.9000	0.9000	1.0000
	F1	0.9333	-	-	-	-	-	0.9333	0.9333	1.0000
yeast-1-4- 5-8_vs_7	Accuracy	0.9567	0.9249	0.9408	0.9278	0.9264	0.8889	0.9567	0.9567	0.9553
	Precision	0.0000	0.1300	0.1905	0.1333	0.0833	0.1205	0.0000	0.0000	0.0000
	Recall	0.0000	0.1333	0.1333	0.1000	0.0667	0.2667	0.0000	0.0000	0.0000
	F1	0.0000	0.1299	0.1504	0.1111	0.0733	0.1654	0.0000	0.0000	0.0000
glass5	Accuracy	0.9767	0.9767	0.9721	0.9767	0.9767	0.9814	0.9721	0.9767	0.9721
	Precision	0.7333	0.5333	0.5333	0.5333	0.5333	0.7333	0.5333	0.7333	0.5333
	Recall	0.6000	0.6000	0.5000	0.6000	0.6000	0.7000	0.5000	0.6000	0.5000
	F1	0.6267	0.5600	0.4933	0.5600	0.5600	0.6933	0.4933	0.6267	0.4933
yeast-2_vs_8	Accuracy	0.9751	0.9606	0.9564	0.9398	0.9585	0.9357	0.9751	0.9792	0.9751
	Precision	0.9500	0.5676	0.4667	0.3000	0.5750	0.3294	0.9500	0.9500	0.9500
	Recall	0.4500	0.5000	0.1500	0.3000	0.5000	0.5500	0.4500	0.5500	0.4500
	F1	0.5614	0.4731	0.2171	0.2889	0.4638	0.3884	0.5614	0.6433	0.5614
yeast4	Accuracy	0.9650	0.9521	0.9555	0.9522	0.9528	0.9178	0.9690	0.9677	0.9656
	Precision	0.3333	0.3268	0.3398	0.3147	0.3331	0.2261	0.7667	0.5667	0.5600
	Recall	0.0782	0.4036	0.3455	0.3836	0.4036	0.5436	0.1564	0.1164	0.1764
	F1	0.1256	0.3561	0.3300	0.3406	0.3556	0.3162	0.2550	0.1884	0.2605
yeast-1-2- 8-9_vs_7	Accuracy	0.9694	0.9504	0.9514	0.9451	0.9462	0.9070	0.9694	0.9715	0.9704
	Precision	0.5000	0.2497	0.2178	0.1582	0.1967	0.0969	0.6167	0.7333	0.6333
	Recall	0.1333	0.2333	0.1667	0.1667	0.2000	0.2333	0.2333	0.2333	0.2333
	F1	0.2071	0.2309	0.1786	0.1599	0.1905	0.1344	0.3249	0.3365	0.3294
yeast5	Accuracy	0.9811	0.9838	0.9825	0.9825	0.9838	0.9805	0.9852	0.9818	0.9852
	Precision	0.8250	0.7060	0.6769	0.6839	0.7060	0.6315	0.7739	0.8100	0.7589
	Recall	0.4917	0.8111	0.8083	0.7861	0.8111	0.8806	0.7222	0.5611	0.7444
	F1	0.5631	0.7400	0.7130	0.7094	0.7400	0.7254	0.7369	0.6212	0.7406
yeast6	Accuracy	0.9818	0.9778	0.9798	0.9771	0.9771	0.9650	0.9845	0.9825	0.9825
	Precision	0.7500	0.5518	0.6083	0.5517	0.5379	0.3870	0.7600	0.7367	0.6795
	Recall	0.3429	0.6000	0.5429	0.6000	0.5714	0.6571	0.4857	0.4000	0.4857
	F1	0.4606	0.5646	0.5601	0.5642	0.5389	0.4761	0.5854	0.5046	0.5550
abalone19	Accuracy	0.9923	0.9744	0.9859	0.9741	0.9751	0.9430	0.9923	0.9919	0.9923
	Precision	0.0000	0.0458	0.0583	0.0467	0.0473	0.0238	0.0000	0.0000	0.0000
	Recall	0.0000	0.1286	0.0619	0.1286	0.1286	0.1524	0.0000	0.0000	0.0000
	F1	0.0000	0.0676	0.0593	0.0684	0.0690	0.0411	0.0000	0.0000	0.0000
cleveland- 0_vs_4	Accuracy	0.9481	0.9711	0.9597	0.9711	0.9654	0.9482	0.9482	0.9482	0.9539
	Precision	0.6000	0.6000	0.5500	0.6000	0.6000	0.5000	0.6000	0.6000	0.6000
	Recall	0.3000	0.6000	0.5333	0.6000	0.5333	0.5333	0.3333	0.3333	0.4000
	F1	0.3933	0.6000	0.5314	0.6000	0.5600	0.4933	0.4000	0.4000	0.4600
ecoli-0- 1_vs_2-3-5	Accuracy	0.9631	0.9509	0.9551	0.9509	0.9509	0.9468	0.9632	0.9590	0.9591
	Precision	0.9100	0.7810	0.8667	0.7833	0.7576	0.7433	0.9200	0.8433	0.8933
	Recall	0.7100	0.7600	0.6800	0.8000	0.8000	0.8000	0.7200	0.7100	0.7200
	F1	0.7798	0.7489	0.7375	0.7720	0.7644	0.7542	0.7798	0.7656	0.7653
ecoli-0- 1_vs_5	Accuracy	0.9750	0.9667	0.9625	0.9708	0.9667	0.9667	0.9667	0.9750	0.9792
	Precision	0.7600	0.8933	0.7333	0.8433	0.8933	0.8267	0.7200	0.7600	0.9600
	Recall	0.7500	0.7500	0.6500	0.8500	0.7500	0.8000	0.7000	0.7500	0.8000
	F1	0.7492	0.7511	0.6648	0.8211	0.7511	0.7854	0.6889	0.7492	0.8444
ecoli-0-1-4- 6_vs_5	Accuracy	0.9786	0.9679	0.9679	0.9679	0.9679	0.9714	0.9714	0.9714	0.9750
	Precision	0.9200	0.7800	0.8167	0.7500	0.7800	0.7900	0.8700	0.8700	0.8700
	Recall	0.8000	0.8000	0.7000	0.8500	0.8000	0.8500	0.7500	0.7500	0.8000
	F1	0.8476	0.7825	0.7500	0.7944	0.7825	0.8167	0.7881	0.7881	0.8167
ecoli-0-1-4- 7_vs_2-3-5-6	Accuracy	0.9643	0.9644	0.9702	0.9644	0.9673	0.9613	0.9643	0.9613	0.9732
	Precision	0.9500	0.8311	0.8600	0.8250	0.8450	0.7726	0.9200	0.8800	0.9000
	Recall	0.6200	0.7867	0.7933	0.7867	0.7867	0.8267	0.6533	0.6533	0.7933
	F1	0.7418	0.7988	0.8212	0.7974	0.8083	0.7913	0.7552	0.7406	0.8358
ecoli-0-1-4- 7_vs_5-6	Accuracy	0.9759	0.9668	0.9698	0.9668	0.9698	0.9638	0.9759	0.9759	0.9820
	Precision	0.9500	0.7700	0.8433	0.7944	0.8033	0.7643	0.9500	0.9500	0.9333
	Recall	0.7200	0.8000	0.7600	0.8400	0.8000	0.8000	0.7200	0.7200	0.8400
	F1	0.8111	0.7806	0.7888	0.7994	0.7988	0.7717	0.8111	0.8111	0.8732
ecoli-0-2- 3-4_vs_5	Accuracy	0.9706	0.9655	0.9705	0.9655	0.9655	0.9655	0.9706	0.9706	0.9754
	Precision	0.8833	0.8600	0.9333	0.8100	0.8600	0.8100	0.8833	0.8833	0.9500
	Recall	0.8000	0.8000	0.7500	0.8500	0.8000	0.8500	0.8000	0.8000	0.8000
	F1	0.8357	0.8206	0.8286	0.8278	0.8206	0.8278	0.8357	0.8357	0.8643
ecoli-0-2- 6-7_vs_3-5	Accuracy	0.9644	0.9510	0.9555	0.9374	0.9555	0.9331	0.9644	0.9600	0.9643
	Precision	0.9000	0.7267	0.7600	0.6533	0.7467	0.6583	0.9000	0.9000	0.8600
	Recall	0.7100	0.7900	0.7000	0.7900	0.7900	0.7900	0.7100	0.6600	0.7400
	F1	0.7786	0.7478	0.7111	0.6985	0.7611	0.7044	0.7786	0.7405	0.7683
ecoli-0-3- 4_vs_5	Accuracy	0.9700	0.9650	0.9750	0.9700	0.9700	0.9600	0.9700	0.9650	0.9800
	Precision	0.8933	0.8600	0.9600	0.8700	0.8600	0.8433	0.8933	0.8833	0.9100
	Recall	0.8000	0.8000	0.8000	0.8500	0.8500	0.8000	0.8000	0.7500	0.9000
	F1	0.8349	0.8206	0.8540	0.8484	0.8492	0.7925	0.8349	0.8071	0.8992
ecoli-0-3- 4-7_vs_5-6	Accuracy	0.9573	0.9532	0.9610	0.9456	0.9532	0.9532	0.9651	0.9651	0.9571
	Precision	0.9200	0.7795	0.8595	0.7478	0.7795	0.7795	0.8833	0.9200	0.8295
	Recall	0.6400	0.7600	0.7600	0.7600	0.7600	0.7600	0.7600	0.7200	0.7600
	F1	0.7343	0.7518	0.7818	0.7280	0.7518	0.7518	0.8066	0.7978	0.7685
ecoli-0-4- 6_vs_5	Accuracy	0.9754	0.9556	0.9655	0.9556	0.9556	0.9606	0.9704	0.9705	0.9704
	Precision	0.9600	0.8167	0.9100	0.7943	0.8167	0.8267	0.9100	0.9200	0.8533
	Recall	0.8000	0.8000	0.7500	0.8500	0.8000	0.8500	0.8000	0.8000	0.9000
	F1	0.8540	0.7748	0.8040	0.7994	0.7748	0.8025	0.8325	0.8317	0.8584
ecoli-0-6- 7_vs_3-5	Accuracy	0.9640	0.9596	0.9506	0.9416	0.9596	0.9415	0.9595	0.9640	0.9640
	Precision	0.9600	0.8433	0.7667	0.7267	0.8433	0.6767	0.9200	0.9600	0.8933
	Recall	0.6800	0.7800	0.7400	0.7800	0.7800	0.7800	0.6800	0.6800	0.7800
	F1	0.7767	0.7867	0.7410	0.7251	0.7867	0.7166	0.7544	0.7767	0.8033
ecoli-0-6- 7_vs_5	Accuracy	0.9727	0.9591	0.9591	0.9591	0.9591	0.9318	0.9773	0.9727	0.9727
	Precision	0.9600	0.8076	0.8033	0.7576	0.8076	0.7043	0.9200	0.9600	0.8933
	Recall	0.7500	0.8500	0.8000	0.9000	0.8500	0.8000	0.8500	0.7500	0.8500
	F1	0.8006	0.7880	0.7456	0.8047	0.7880	0.6675	0.8603	0.8006	0.8425
glass-0-1- 4-6_vs_2	Accuracy	0.9122	0.8780	0.8732	0.9073	0.8780	0.8146	0.9171	0.9073	0.9024
	Precision	0.0000	0.3733	0.3590	0.5033	0.3733	0.2591	0.1500	0.0000	0.2333
	Recall	0.0000	0.3833	0.3833	0.4333	0.3833	0.5000	0.2000	0.0000	0.2000
	F1	0.0000	0.3078	0.2944	0.4110	0.3078	0.3038	0.1714	0.0000	0.2133
glass-0-1- 5_vs_2	Accuracy	0.9015	0.8429	0.8486	0.8429	0.8429	0.7787	0.9129	0.9013	0.9072
	Precision	0.2000	0.1905	0.2833	0.2500	0.1905	0.1651	0.6333	0.2000	0.6000
	Recall	0.0667	0.2333	0.2833	0.2833	0.2333	0.3500	0.2833	0.0667	0.2333
	F1	0.1000	0.1943	0.2786	0.2643	0.1943	0.2224	0.3810	0.1000	0.3333
glass-0-4_vs_5	All	1.0000	1.0000	1.0000	1.0000	1.0000	1.0000	1.0000	1.0000	1.0000
glass-0- 6_vs_5	Accuracy	0.9905	0.9818	0.9723	0.9909	0.9818	0.9909	0.9905	0.9905	0.9905
	Precision	0.8000	0.9333	0.7333	1.0000	0.9333	1.0000	0.8000	0.8000	0.8000
	Recall	0.8000	0.9000	0.7000	0.9000	0.9000	0.9000	0.8000	0.8000	0.8000
	F1	0.8000	0.8933	0.6933	0.9333	0.8933	0.9333	0.8000	0.8000	0.8000
led7digit- 0-2-4-5-6- 7-8-9_vs_1	Accuracy	0.9662	0.9684	0.9662	0.9617	0.9684	0.9324	0.9662	0.9662	0.9594
	Precision	0.7984	0.8012	0.7984	0.7634	0.8012	0.8667	0.7984	0.7984	0.7523
	Recall	0.8071	0.8321	0.8071	0.8071	0.8321	0.3000	0.8071	0.8071	0.8071
	F1	0.8005	0.8137	0.8005	0.7811	0.8137	0.4215	0.8005	0.8005	0.7737
yeast-0-2- 5-6_vs_3-7- 8-9	Accuracy	0.9382	0.9293	0.9283	0.9193	0.9293	0.9044	0.9372	0.9373	0.9323
	Precision	0.8143	0.6496	0.6662	0.5982	0.6535	0.5172	0.7682	0.7790	0.7099
	Recall	0.4842	0.6353	0.5653	0.6053	0.6253	0.6958	0.5353	0.5147	0.5447
	F1	0.6030	0.6373	0.6067	0.5959	0.6342	0.5905	0.6261	0.6168	0.6129
yeast-0-2- 5-7-9_vs_3- 6-8	Accuracy	0.9641	0.9592	0.9651	0.9572	0.9582	0.9532	0.9631	0.9661	0.9641
	Precision	0.8587	0.7837	0.8588	0.7686	0.7807	0.7266	0.8439	0.8584	0.8328
	Recall	0.7695	0.8195	0.7889	0.8295	0.8195	0.8500	0.7795	0.8000	0.8095
	F1	0.8066	0.7982	0.8165	0.7931	0.7952	0.7808	0.8049	0.8213	0.8159
yeast-0-3-5- 9_vs_7-8	Accuracy	0.9111	0.8775	0.8853	0.8794	0.8873	0.8182	0.9072	0.9151	0.9131
	Precision	0.6333	0.3875	0.4095	0.4195	0.4389	0.3046	0.4933	0.6333	0.5950
	Recall	0.1800	0.4600	0.3600	0.4600	0.4800	0.6200	0.2400	0.2400	0.3200
	F1	0.2620	0.4099	0.3739	0.4241	0.4472	0.4045	0.3150	0.3381	0.4076
abalone- 3_vs_11	Accuracy	0.9980	0.9980	0.9980	0.9980	0.9980	0.9980	0.9980	1.0000	0.9940
	Precision	0.9500	0.9500	0.9500	0.9500	0.9500	0.9500	0.9500	1.0000	0.9000
	Recall	1.0000	1.0000	1.0000	1.0000	1.0000	1.0000	1.0000	1.0000	1.0000
	F1	0.9714	0.9714	0.9714	0.9714	0.9714	0.9714	0.9714	1.0000	0.9333
abalone- 17_vs_7-8- 9-10	Accuracy	0.9756	0.9577	0.9615	0.9568	0.9564	0.9397	0.9765	0.9748	0.9748
	Precision	0.6333	0.2873	0.3054	0.2907	0.2811	0.2431	0.5667	0.3667	0.5100
	Recall	0.1045	0.4470	0.4136	0.4303	0.4485	0.5864	0.1591	0.0515	0.1561
	F1	0.1761	0.3451	0.3460	0.3354	0.3427	0.3360	0.2457	0.0886	0.2349
abalone- 19_vs_10- 11-12-13	Accuracy	0.9803	0.9427	0.9605	0.9464	0.9427	0.8964	0.9803	0.9803	0.9803
	Precision	0.0000	0.0926	0.0889	0.0860	0.0671	0.0622	0.0000	0.0000	0.0000
	Recall	0.0000	0.2238	0.1286	0.2000	0.1667	0.3238	0.0000	0.0000	0.0000
	F1	0.0000	0.1302	0.1028	0.1199	0.0955	0.1037	0.0000	0.0000	0.0000
abalone- 20_vs_8-9-10	Accuracy	0.9859	0.9734	0.9786	0.9723	0.9708	0.9640	0.9849	0.9859	0.9854
	Precision	0.0000	0.2271	0.2885	0.2253	0.1833	0.1775	0.0000	0.0000	0.0000
	Recall	0.0000	0.4800	0.4333	0.4800	0.4000	0.5133	0.0000	0.0000	0.0000
	F1	0.0000	0.3051	0.3438	0.3034	0.2493	0.2624	0.0000	0.0000	0.0000
abalone- 21_vs_8	Accuracy	0.9776	0.9690	0.9776	0.9638	0.9690	0.9621	0.9759	0.9776	0.9794
	Precision	0.3333	0.5083	0.6033	0.3917	0.5083	0.3488	0.3000	0.3333	0.5500
	Recall	0.2000	0.5667	0.5667	0.5667	0.5667	0.6667	0.1333	0.2000	0.3333
	F1	0.2333	0.4662	0.5300	0.4335	0.4662	0.4361	0.1800	0.2333	0.3714
car-good	Accuracy	0.9491	0.9479	0.9485	0.9502	0.9479	0.9346	0.9497	0.9502	0.9485
	Precision	0.3228	0.3549	0.3594	0.3717	0.3549	0.3535	0.3611	0.3425	0.3363
	Recall	0.2473	0.3187	0.3330	0.3341	0.3187	0.7516	0.3187	0.2615	0.2747
	F1	0.2780	0.3287	0.3396	0.3485	0.3287	0.4782	0.3352	0.2919	0.2996
car-vgood	Accuracy	0.9618	0.9595	0.9601	0.9601	0.9595	0.9612	0.9618	0.9606	0.9589
	Precision	0.5102	0.4585	0.4776	0.4776	0.4585	0.4954	0.5016	0.4969	0.4734
	Recall	0.3385	0.3385	0.3538	0.3538	0.3385	0.7846	0.3385	0.3231	0.3692
	F1	0.3913	0.3812	0.3991	0.3991	0.3812	0.6055	0.3907	0.3771	0.4006
dermatology-6	All	1.0000	1.0000	1.0000	1.0000	1.0000	1.0000	1.0000	1.0000	1.0000
flare-F	Accuracy	0.9503	0.9381	0.9390	0.9353	0.9400	0.9156	0.9475	0.9493	0.9437
	Precision	0.1333	0.1471	0.1650	0.1379	0.1789	0.2449	0.1250	0.1550	0.1817
	Recall	0.0694	0.1417	0.1667	0.1417	0.1917	0.5139	0.0694	0.0944	0.1667
	F1	0.0908	0.1421	0.1638	0.1364	0.1829	0.3299	0.0876	0.1158	0.1705
kddcup- buffer_overflow_ vs_back	All	1.0000	1.0000	1.0000	1.0000	1.0000	1.0000	1.0000	1.0000	1.0000
kddcup- guess_passwd_ vs_satan	All	1.0000	1.0000	1.0000	-	1.0000	1.0000	1.0000	1.0000	1.0000
kddcup- land_vs_ portsweep	All	1.0000	1.0000	1.0000	-	1.0000	1.0000	1.0000	0.9991	1.0000
kr-vs-k- one_vs_fifteen	All	1.0000	1.0000	1.0000	1.0000	1.0000	1.0000	1.0000	1.0000	1.0000
kr-vs-k-zero- one_vs_draw	Accuracy	0.9959	0.9959	0.9955	0.9962	0.9966	0.9945	0.9976	0.9966	0.9983
	Precision	0.9795	0.9445	0.9609	0.9437	0.9623	0.8882	0.9810	0.9905	0.9814
	Recall	0.9048	0.9429	0.9143	0.9524	0.9429	0.9714	0.9524	0.9143	0.9714
	F1	0.9400	0.9428	0.9366	0.9477	0.9519	0.9274	0.9661	0.9502	0.9761
lymphography- normal- fibrosis	Accuracy	0.9864	-	-	-	-	-	0.9864	0.9864	0.9864
	Precision	0.6000	-	-	-	-	-	0.6000	0.6000	0.6000
	Recall	0.6000	-	-	-	-	-	0.6000	0.6000	0.6000
	F1	0.6000	-	-	-	-	-	0.6000	0.6000	0.6000
poker-8_vs_6	Accuracy	0.9885	0.9946	0.9932	0.9939	0.9946	0.9946	0.9885	0.9885	0.9885
	Precision	0.0000	0.8000	0.6000	0.8000	0.8000	0.8000	-	-	-
	Recall	0.0000	0.5500	0.4333	0.4833	0.5500	0.5500	-	-	-
	F1	0.0000	0.6133	0.4933	0.5733	0.6133	0.6133	-	-	-
poker-8-9_vs_5	Accuracy	0.9880	0.9855	0.9870	0.9860	0.9855	0.9827	0.9880	0.9880	0.9880
	Precision	0.0000	0.0000	0.0000	0.0000	0.0000	0.0000	0.0000	0.0000	0.0000
	Recall	0.0000	0.0000	0.0000	0.0000	0.0000	0.0000	0.0000	0.0000	0.0000
	F1	0.0000	0.0000	0.0000	0.0000	0.0000	0.0000	0.0000	0.0000	0.0000
poker-9_vs_7	Accuracy	0.9713	0.9713	0.9673	0.9754	0.9713	0.9672	0.9713	0.9713	0.9713
	Precision	0.2000	0.2000	0.0000	0.4000	0.2000	0.2000	0.2000	0.2000	0.2000
	Recall	0.2000	0.1000	0.0000	0.2000	0.1000	0.1000	0.2000	0.2000	0.2000
	F1	0.2000	0.1333	0.0000	0.2667	0.1333	0.1333	0.2000	0.2000	0.2000
shuttle-2_vs_5	All	1.0000	1.0000	1.0000	1.0000	1.0000	1.0000	1.0000	1.0000	1.0000
shuttle-6_vs_2-3	All	1.0000	1.0000	1.0000	1.0000	1.0000	1.0000	1.0000	1.0000	1.0000
winequality- red-3_vs_5	Accuracy	0.9855	0.9653	0.9768	0.9667	0.9667	0.9566	0.9855	0.9855	0.9855
	Precision	-	-	-	-	-	-	-	-	-
	Recall	-	-	-	-	-	-	-	-	-
	F1	-	-	-	-	-	-	-	-	-
winequality- red-4	Accuracy	0.9669	0.9475	0.9600	0.9475	0.9481	0.9137	0.9662	0.9669	0.9656
	Precision	0.1000	0.1831	0.0667	0.1754	0.1458	0.1268	0.0000	0.1000	0.0000
	Recall	0.0182	0.1691	0.0200	0.1327	0.1127	0.2618	0.0000	0.0182	0.0000
	F1	0.0308	0.1748	0.0308	0.1473	0.1268	0.1697	0.0000	0.0308	0.0000
winequality- red-8_vs_6-7	Accuracy	0.9813	0.9602	0.9801	0.9602	0.9614	0.9216	0.9813	0.9813	0.9813
	Precision	0.4000	0.1333	0.4000	0.1333	0.1467	0.1103	0.4000	0.4000	0.4000
	Recall	0.1000	0.2000	0.1000	0.2000	0.2000	0.3833	0.1000	0.1000	0.1000
	F1	0.1600	0.1600	0.1600	0.1600	0.1689	0.1700	0.1600	0.1600	0.1600
winequality- red-8_vs_6	Accuracy	0.9741	0.9543	0.9726	0.9527	0.9481	0.9238	0.9741	0.9726	0.9726
	Precision	0.4000	0.2444	0.3667	0.2305	0.2133	0.1446	0.3000	0.3000	0.3000
	Recall	0.1167	0.3000	0.1667	0.3000	0.3000	0.4167	0.1167	0.1167	0.1167
	F1	0.1800	0.2476	0.2133	0.2443	0.2258	0.2062	0.1600	0.1600	0.1600
winequality- white-3_vs_7	Accuracy	0.9811	0.9711	0.9778	0.9700	0.9678	0.9478	0.9811	0.9800	0.9800
	Precision	0.4000	0.1667	0.3000	0.1667	0.1286	0.1812	0.6000	0.4000	0.6000
	Recall	0.1500	0.1500	0.1500	0.1500	0.1000	0.4000	0.2000	0.1500	0.2000
	F1	0.2133	0.1467	0.1800	0.1467	0.1030	0.2457	0.2933	0.2133	0.2933
winequality- white-3-9_vs_5	Accuracy	0.9825	0.9730	0.9784	0.9717	0.9723	0.9575	0.9825	0.9825	0.9818
	Precision	0.2000	0.1119	0.2500	0.0833	0.1067	0.0993	0.2000	0.2000	0.2000
	Recall	0.0400	0.1200	0.0800	0.0800	0.1200	0.2000	0.0400	0.0400	0.0400
	F1	0.0667	0.1141	0.1111	0.0808	0.1127	0.1307	0.0667	0.0667	0.0667
winequality- white-9_vs_4	Accuracy	0.9763	-	-	-	-	-	0.9763	0.9763	0.9763
	Precision	0.2000	-	-	-	-	-	0.2000	0.2000	0.2000
	Recall	0.2000	-	-	-	-	-	0.2000	0.2000	0.2000
	F1	0.2000	-	-	-	-	-	0.2000	0.2000	0.2000
zoo-3	Accuracy	0.9705	-	-	-	-	-	0.9705	0.9705	0.9705
	Precision	0.4000	-	-	-	-	-	0.4000	0.4000	0.4000
	Recall	0.4000	-	-	-	-	-	0.4000	0.4000	0.4000
	F1	0.4000	-	-	-	-	-	0.4000	0.4000	0.4000

## Appendix D

**Table A4:** Full KEEL benchmark results (accuracy, precision, recall, and F1) for all noisy/borderline datasets. Best results in bold.

Dataset	Metric	Original	SMOTE	Borderline- SMOTE	ADASYN	SMOTE +Tomek	SMOTEENN	Radius-SMOTE	WCOM KKNB	WISEST (Ours)
03subcl5- 600-5-0-BI	Accuracy	0.9483	0.9467	0.9450	0.9483	0.9450	0.9233	0.9400	**0.9533**	0.9483
	Precision	0.8378	0.8235	0.8131	0.8181	0.8176	0.7263	0.7981	**0.8771**	0.7810
	Recall	0.8600	0.8700	0.8900	0.9000	0.8700	0.8800	0.8700	0.8400	**0.9700**
	F1	0.8457	0.8430	0.8454	0.8542	0.8403	0.7940	0.8292	0.8551	**0.8641**
03subcl5- 600-5-30-BI	Accuracy	0.8850	0.8517	0.8483	0.8350	0.8367	0.8350	0.8700	**0.8917**	0.8667
	Precision	0.7052	0.5447	0.5363	0.5056	0.5153	0.5070	0.6017	**0.7222**	0.5890
	Recall	0.5700	0.7400	0.7600	0.7600	0.7000	**0.8100**	0.6600	0.6000	0.7000
	F1	0.6263	0.6250	0.6278	0.6055	0.5906	0.6227	0.6289	0.6521	**0.6387**
03subcl5- 600-5-50-BI	Accuracy	0.8600	0.8150	0.8150	0.8100	0.8100	0.7800	0.8350	**0.8583**	0.8350
	Precision	0.6245	0.4730	0.4692	0.4582	0.4606	0.4242	0.5143	**0.6156**	0.5125
	Recall	0.4600	0.6800	0.6800	0.7100	0.6700	**0.8300**	0.5200	0.4600	0.5800
	F1	0.5278	0.5542	0.5536	0.5561	0.5433	0.5591	0.5157	0.5242	**0.5432**
03subcl5- 600-5-60-BI	Accuracy	0.8350	0.8050	0.7933	0.8033	0.8033	0.7850	0.8050	**0.8333**	0.8000
	Precision	0.5150	0.4436	0.4246	0.4451	0.4384	0.4250	0.4185	**0.4992**	0.4201
	Recall	0.3700	0.6500	0.6300	0.7100	0.6300	**0.8100**	0.4600	0.3500	0.5400
	F1	0.4243	0.5259	0.5054	0.5458	0.5161	0.5572	0.4369	0.4102	**0.4710**
03subcl5- 600-5-70-BI	Accuracy	0.8367	0.8050	0.7917	0.7967	0.8133	0.7700	0.8100	**0.8400**	0.7983
	Precision	0.5442	0.4516	0.4267	0.4431	0.4667	0.4066	0.4371	**0.5273**	0.4177
	Recall	0.3500	0.7300	0.6600	**0.7900**	0.7600	0.8000	0.4600	0.3700	0.4800
	F1	0.4142	0.5565	0.5163	**0.5657**	0.5764	0.5381	0.4443	0.4315	0.4415
03subcl5- 800-7-0-BI	Accuracy	0.9538	0.9525	0.9538	**0.9575**	0.9550	0.9462	0.9550	0.9563	0.9550
	Precision	0.8212	0.7744	0.7896	0.7963	0.7878	0.7274	0.7920	**0.8560**	0.7662
	Recall	0.8100	0.8800	0.8700	0.8900	0.8800	0.9100	0.8700	0.7800	**0.9300**
	F1	0.8149	0.8224	0.8235	**0.8391**	0.8297	0.8067	0.8283	0.8155	0.8387
03subcl5- 800-7-30-BI	Accuracy	**0.9113**	0.8613	0.8588	0.8575	0.8575	0.8263	0.9025	0.9088	0.9025
	Precision	**0.6994**	0.4647	0.4618	0.4707	0.4552	0.4012	0.6079	0.6919	0.6117
	Recall	0.5400	0.6900	0.6900	**0.7400**	0.6800	**0.7400**	0.6300	0.5200	0.6600
	F1	0.5990	0.5537	0.5514	0.5707	0.5434	0.5185	0.6149	0.5873	**0.6324**
03subcl5- 800-7-50-BI	Accuracy	0.8838	0.8200	0.8313	0.8138	0.8088	0.7713	0.8738	**0.8863**	0.8625
	Precision	0.5883	0.3751	0.4040	0.3688	0.3604	0.3180	0.4953	**0.6070**	0.4525
	Recall	0.3400	0.6400	0.6800	0.6700	0.6400	0.7200	0.4700	0.3300	**0.4800**
	F1	0.4246	0.4720	0.5054	0.4740	0.4590	0.4401	0.4820	0.4244	**0.4650**
03subcl5- 800-7-60-BI	Accuracy	0.8787	0.8200	0.8200	0.8088	0.8088	0.7875	0.8588	**0.8750**	0.8412
	Precision	0.5383	0.3747	0.3684	0.3624	0.3566	0.3371	0.4355	**0.5110**	0.3693
	Recall	0.3000	0.6100	0.5700	**0.6700**	0.6400	0.7300	0.3800	0.2800	0.3700
	F1	0.3780	0.4617	0.4454	**0.4691**	0.4572	0.4607	0.4009	0.3573	0.3636
03subcl5- 800-7-70-BI	Accuracy	0.8600	0.8050	0.8038	0.8000	0.7925	0.7513	0.8463	**0.8600**	0.8412
	Precision	0.4565	0.3461	0.3426	0.3477	0.3293	0.3028	0.3871	**0.4412**	0.3815
	Recall	0.2500	0.6300	0.6100	**0.6800**	0.6300	0.7300	0.3900	0.2100	0.3900
	F1	0.3082	0.4466	0.4380	**0.4597**	0.4318	0.4263	0.3838	0.2737	0.3836
04clover5z- 600-5-0-BI	Accuracy	0.9200	0.9150	0.9200	0.9083	0.9200	0.8967	0.9150	**0.9350**	0.9150
	Precision	0.7802	0.7181	0.7166	0.6819	0.7235	0.6446	0.7256	**0.8438**	0.6931
	Recall	0.7400	0.8300	0.8700	0.8700	0.8700	0.8900	0.8100	0.7600	**0.9000**
	F1	0.7559	0.7683	0.7844	0.7625	0.7867	0.7451	0.7623	**0.7969**	0.7816
04clover5z- 600-5-30-BI	Accuracy	0.8717	0.8667	0.8600	0.8600	0.8600	0.8283	0.8683	0.8700	**0.8700**
	Precision	0.6573	0.5875	0.5672	0.5615	0.5690	0.5011	0.6067	**0.6446**	0.5955
	Recall	0.4900	0.7200	0.7300	0.7500	0.7100	**0.8000**	0.6300	0.5100	0.7100
	F1	0.5569	0.6438	0.6365	0.6406	0.6294	0.6113	0.6113	0.5642	**0.6451**
04clover5z- 600-5-50-BI	Accuracy	0.8433	0.8350	0.8367	**0.8533**	0.8350	0.8050	0.8517	0.8450	0.8417
	Precision	0.5559	0.5155	0.5126	0.5487	0.5179	0.4613	0.5598	0.5535	**0.5259**
	Recall	0.3500	0.6900	0.7000	**0.7800**	0.6900	0.8200	0.5500	0.3600	0.5800
	F1	0.4249	0.5851	0.5903	**0.6421**	0.5849	0.5880	0.5484	0.4322	0.5480
04clover5z- 600-5-60-BI	Accuracy	0.8217	0.8117	0.7983	0.8000	0.8050	0.7650	0.8117	**0.8267**	0.8067
	Precision	0.4308	0.4594	0.4368	0.4401	0.4471	0.3935	0.4472	**0.4615**	0.4315
	Recall	0.3000	0.6600	0.6300	0.6600	0.6500	**0.7400**	0.4700	0.3400	0.4900
	F1	0.3497	0.5397	0.5133	0.5266	0.5270	0.5121	0.4542	0.3877	**0.4557**
04clover5z- 600-5-70-BI	Accuracy	0.8333	0.8133	0.7983	0.8017	0.8133	0.7683	0.8233	0.8200	**0.8083**
	Precision	0.5237	0.4651	0.4263	0.4379	0.4660	0.4093	0.4781	**0.4765**	0.4309
	Recall	0.3200	0.6600	0.6000	0.6400	**0.7100**	0.7800	0.4700	0.3100	0.4700
	F1	0.3874	0.5417	0.4953	0.5181	**0.5583**	0.5318	0.4687	0.3653	0.4442
04clover5z- 800-7-0-BI	Accuracy	0.9488	0.9463	**0.9550**	0.9450	0.9487	0.9200	0.9450	0.9450	0.9325
	Precision	0.8775	0.7442	0.7918	0.7412	0.7609	0.6282	0.7932	**0.8800**	0.6957
	Recall	0.6900	0.8700	**0.8800**	0.8600	0.8600	0.8900	0.7600	0.6500	0.8200
	F1	0.7693	0.8012	**0.8302**	0.7951	0.8067	0.7356	0.7741	0.7458	0.7513
04clover5z- 800-7-30-BI	Accuracy	0.9100	0.8862	0.8813	0.8825	0.8825	0.8575	0.9000	**0.9075**	0.8975
	Precision	0.7319	0.5369	0.5192	0.5232	0.5245	0.4568	0.6227	**0.7230**	0.5958
	Recall	0.4400	0.7000	0.7200	0.7000	0.6900	**0.7500**	0.5300	0.4300	0.5800
	F1	0.5489	0.6060	0.6025	0.5977	0.5948	0.5669	0.5697	0.5375	**0.5857**
04clover5z- 800-7-50-BI	Accuracy	0.8738	0.8338	0.8375	0.8363	0.8313	0.8087	0.8700	**0.8713**	0.8500
	Precision	0.4995	0.3987	0.4008	0.4116	0.3910	0.3690	0.4781	**0.4605**	0.3998
	Recall	0.2400	0.6200	0.5700	**0.6800**	0.6200	0.7300	0.4100	0.2000	0.3900
	F1	0.3223	0.4829	0.4685	**0.5116**	0.4784	0.4891	0.4397	0.2768	0.3915
04clover5z- 800-7-60-BI	Accuracy	0.8513	0.8212	0.8325	0.8275	0.8250	0.8025	0.8425	**0.8575**	0.8363
	Precision	0.3521	0.3839	0.4000	0.3992	0.3892	0.3678	0.3726	**0.3919**	0.3566
	Recall	0.2000	0.6200	0.6100	**0.6500**	0.6200	0.7400	0.3300	0.2200	0.3600
	F1	0.2519	0.4718	0.4819	**0.4920**	0.4756	0.4895	0.3434	0.2733	0.3556
04clover5z- 800-7-70-BI	Accuracy	0.8687	0.8237	0.8200	0.8262	0.8188	0.7863	0.8525	**0.8700**	0.8525
	Precision	0.4546	0.3656	0.3553	0.3801	0.3640	0.3414	0.4055	**0.4883**	0.4055
	Recall	0.2300	0.5900	0.5500	**0.6400**	0.6200	0.7500	0.3700	0.2000	0.3900
	F1	0.3008	0.4495	0.4313	**0.4751**	0.4571	0.4677	0.3843	0.2809	0.3972
paw02a- 600-5-0-BI	Accuracy	0.9700	0.9667	0.9717	**0.9733**	0.9700	0.9600	0.9700	0.9700	0.9650
	Precision	0.9132	0.8714	0.9012	0.8909	0.8829	0.8344	0.8904	**0.9132**	0.8594
	Recall	0.9100	0.9400	0.9400	**0.9600**	0.9500	0.9500	0.9400	0.9100	**0.9500**
	F1	0.9104	0.9032	0.9185	**0.9233**	0.9132	0.8879	0.9125	0.9104	0.9011
paw02a- 600-5-30-BI	Accuracy	0.9283	0.9033	0.8983	0.8883	0.8967	0.8750	0.9133	**0.9300**	0.8967
	Precision	0.8821	0.6884	0.6698	0.6300	0.6673	0.6048	0.7552	**0.8754**	0.6753
	Recall	0.6900	0.8100	0.8100	0.8600	0.8200	0.8400	0.7600	0.7100	**0.8200**
	F1	0.7586	0.7378	0.7261	0.7231	0.7291	0.6958	0.7444	**0.7688**	0.7290
paw02a- 600-5-50-BI	Accuracy	0.8900	0.8700	0.8633	0.8583	0.8617	0.8467	0.8800	**0.8983**	0.8733
	Precision	0.7153	0.5907	0.5768	0.5573	0.5675	0.5292	0.6304	**0.7529**	0.6142
	Recall	0.5800	0.7600	0.7700	0.7900	0.7600	0.8000	0.6900	0.5900	**0.7000**
	F1	0.6342	0.6601	0.6527	0.6495	0.6465	0.6349	0.6556	**0.6555**	0.6490
paw02a- 600-5-60-BI	Accuracy	0.8683	0.8350	0.8283	0.8317	0.8400	0.8083	0.8450	**0.8617**	0.8583
	Precision	0.6249	0.5043	0.4898	0.4966	0.5143	0.4548	0.5302	**0.6040**	0.5711
	Recall	0.5300	0.7300	0.7200	0.7800	0.7500	0.7500	0.5900	0.5200	**0.6300**
	F1	0.5693	0.5953	0.5823	0.6059	0.6095	0.5651	0.5577	0.5529	**0.5964**
paw02a- 600-5-70-BI	Accuracy	0.8683	0.8300	0.8333	0.8233	0.8250	0.8200	0.8350	**0.8667**	0.8350
	Precision	0.6204	0.4959	0.5017	0.4823	0.4870	0.4792	0.5041	**0.6129**	0.5068
	Recall	0.5400	0.7200	0.7200	0.7200	0.7500	**0.8200**	0.6500	0.5400	0.6600
	F1	0.5756	0.5855	0.5895	0.5759	0.5888	**0.6038**	0.5671	0.5733	0.5720
paw02a- 800-7-0-BI	Accuracy	0.9712	0.9688	**0.9737**	0.9700	0.9688	0.9650	0.9700	0.9713	0.9675
	Precision	0.8884	0.8417	0.8793	0.8449	0.8417	0.8072	0.8592	**0.8920**	0.8285
	Recall	0.8900	0.9300	0.9200	**0.9400**	0.9300	**0.9500**	0.9200	0.8900	**0.9500**
	F1	0.8864	0.8820	**0.8981**	0.8879	0.8820	0.8717	0.8856	0.8870	0.8816
paw02a- 800-7-30-BI	Accuracy	0.9463	0.8988	0.9113	0.8863	0.9000	0.8763	0.9375	**0.9463**	0.9250
	Precision	0.8770	0.5720	0.6241	0.5325	0.5824	0.5012	0.7749	**0.8720**	0.6850
	Recall	0.6600	0.7700	0.7800	0.8100	0.7500	0.8300	0.7100	0.6700	**0.7400**
	F1	0.7483	0.6541	0.6882	0.6412	0.6516	0.6246	0.7355	**0.7498**	0.7105
paw02a- 800-7-50-BI	Accuracy	0.9238	0.8600	0.8575	0.8575	0.8650	0.8463	0.9025	**0.9250**	0.8900
	Precision	0.7890	0.4611	0.4529	0.4566	0.4754	0.4393	0.6134	**0.7741**	0.5553
	Recall	0.5300	0.7200	0.6800	0.7400	0.7300	0.8100	0.6200	0.5600	**0.6500**
	F1	0.6328	0.5616	0.5419	0.5645	0.5749	0.5691	0.6127	**0.6467**	0.5961
paw02a- 800-7-60-BI	Accuracy	0.9000	0.8538	0.8575	0.8413	0.8538	0.8175	0.8913	**0.8988**	0.8787
	Precision	0.6464	0.4474	0.4621	0.4259	0.4476	0.3856	0.5841	**0.6487**	0.5283
	Recall	0.4700	0.7000	0.7000	0.7300	0.7100	0.7700	0.5500	0.4700	**0.5400**
	F1	0.5363	0.5452	0.5553	0.5368	0.5481	0.5131	0.5607	0.5359	**0.5292**
paw02a- 800-7-70-BI	Accuracy	0.8888	0.8513	0.8563	0.8375	0.8475	0.8125	0.8787	**0.8800**	0.8663
